# Zbp1-positive cells are osteogenic progenitors in periodontal ligament

**DOI:** 10.1038/s41598-021-87016-1

**Published:** 2021-04-06

**Authors:** Tsugumi Ueda, Tomoaki Iwayama, Kiwako Tomita, Shuji Matsumoto, Mizuho Iwashita, Phan Bhongsatiern, Hiromi Sakashita, Chiharu Fujihara, Masahide Takedachi, Shinya Murakami

**Affiliations:** Department of Periodontology, Osaka University Graduate School of Dentistry, Suita, Osaka Japan

**Keywords:** Cell biology, Periodontics

## Abstract

Periodontal ligament (PDL) possesses a stem/progenitor population to maintain the homeostasis of periodontal tissue. However, transcription factors that regulate this population have not yet been identified. Thus, we aimed to identify a molecule related to the osteogenic differentiation of PDL progenitors using a single cell-based strategy in this study. We first devised a new protocol to isolate PDL cells from the surface of adult murine molars and established 35 new single cell-derived clones from the PDL explant. Among these clones, six clones with high (high clones, n = 3) and low (low clones, n = 3) osteogenic potential were selected. Despite a clear difference in the osteogenic potential of these clones, no significant differences in their cell morphology, progenitor cell marker expression, alkaline phosphatase activity, proliferation rate, and differentiation-related gene and protein expression were observed. RNA-seq analysis of these clones revealed that *Z-DNA binding protein-1* (*Zbp1*) was significantly expressed in the high osteogenic clones, indicating that Zbp1 could be a possible marker and regulator of the osteogenic differentiation of PDL progenitor cells. Zbp1-positive cells were distributed sparsely throughout the PDL. In vitro Zbp1 expression in the PDL clones remained at a high level during osteogenic differentiation. The CRISPR/Cas9 mediated *Zbp1* knockout in the high clones resulted in a delay in cell differentiation. On the other hand, *Zbp1* overexpression in the low clones promoted cell differentiation. These findings suggested that Zbp1 marked the PDL progenitors with high osteogenic potential and promoted their osteogenic differentiation. Clarifying the mechanism of differentiation of PDL cells by Zbp1 and other factors in future studies will facilitate a better understanding of periodontal tissue homeostasis and repair, possibly leading to the development of novel therapeutic measures.

## Introduction

Stem/progenitor cells in the periodontal ligament (PDL) play a central role in the maintenance of periodontal tissue homeostasis by giving rise to osteoblasts/cementoblasts and periodontal fibroblasts^1^. Indeed, cells isolated from the PDL have been shown to possess the potential of multi-differentiation into these cell types^2^. Additionally, in vivo evidence suggested that injury-responsive cells originate from the PDL^3–5^. However, since the progenitor cell population of adult PDL is highly heterogeneous, it has not yet been defined by specific markers. Furthermore, the regulation of this population remains to be elucidated.

To answer these questions, a comparison of PDL cells with high and low osteogenic potential and the identification of a molecule that can define the osteogenic potential of these cells can be valuable. Since the prospective isolation of PDL progenitors from adult mice has remained infeasible to date, we aimed to perform functional analysis in vitro at a clonal level to better understand these PDL progenitor cells. The explant method has been successfully used for young mice in which contamination of developmental cells is likely to occur^6^. Other groups have established adult PDL cell lines by immortalizing them by introducing SV-40 T antigen or harvesting cells from ImmortoMouse^7^. However, no study on the primary culture of adult murine PDL cells without induction of transformation has been reported. It has been shown that stem/progenitor cells can be cultured without the loss of their differentiation potential using basic fibroblast growth factor (FGF-2)^8,9^. Interestingly, rat PDL cells can be expanded without losing their multipotency in a neurosphere-forming culture containing FGF-2 and the other cytokines^10^. In this study, we developed a primary culture protocol to isolate and culture adult PDL cells using FGF-2 without transformation and established a number of single-cell-derived PDL cell clones with or without osteogenic potential.

Among the factors known to regulate osteogenic differentiation, runt-related transcription factor 2 (Runx2) and Sp7/Osterix are essential transcription regulators of osteoblast differentiation^11–13^. It has been reported that the onset of Runx2 expression occurs before bone formation^14^, suggesting that cofactors other than Runx2 may be involved^15^. Furthermore, immunohistochemical studies have shown that the majority of PDL cells consistently express Runx2^16^, indicating the presence of other cofactors. If molecules that regulate osteogenic differentiation can be identified, PDL stem/progenitor cell differentiation can be controlled. Additionally, it will facilitate a better understanding of the molecular basis of periodontal tissue regeneration. The identification of molecular markers that specifically label the progenitor cells may lead to the identification of various PDL cell populations. Furthermore, in vivo characterization of the localization of these cell groups will potentially elucidate the functioning of PDL cells.

Thus, in this study, we established single cell-derived clones that exhibited high (high clones) and low (low clones) osteogenic potential and assessed their cellular properties. We identified differentiation regulators by comprehensively comparing gene expression and the molecules involved in osteogenic differentiation of PDL progenitor cells by performing lentivirus- and CRISPR/Cas9-based functional experiments to identify the progenitor cells retrospectively.

## Results

### Isolation of murine PDL cells

**Figure 1:**
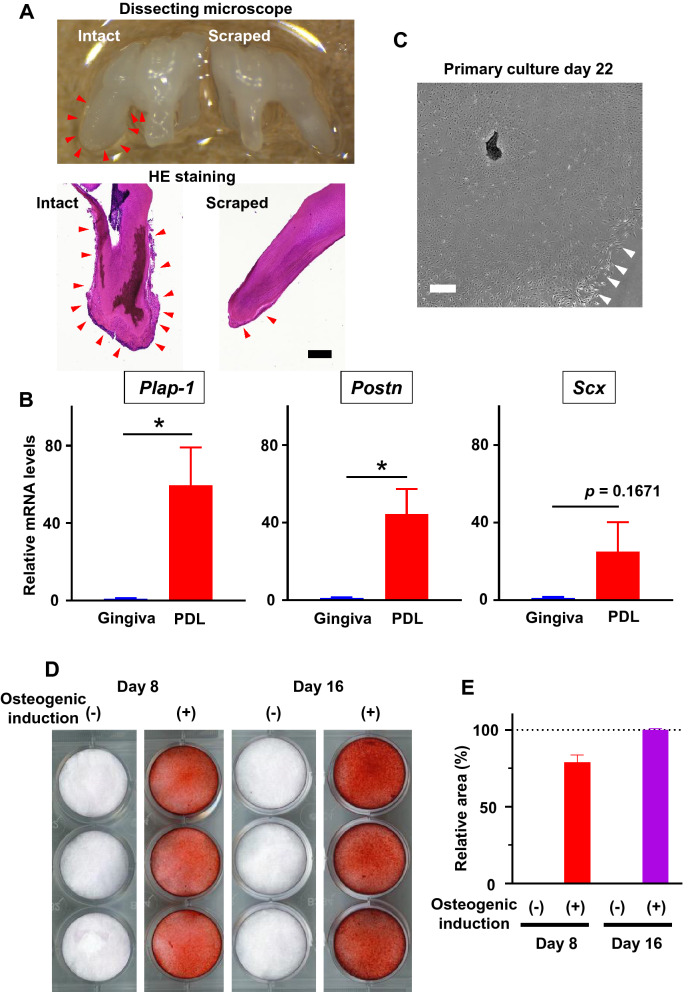
Isolation of murine periodontal ligament (PDL) cells with osteogenic potential. (**A**) The intact (left) or PDL-scraped (right) M1 teeth were imaged with dissecting microscope. The same teeth were histologically analyzed, and HE staining images were shown. Red arrowheads indicate the remaining PDL. Scale bar: 200 μm. (**B**) The mRNA expression levels of PDL markers *Plap-1/Aspn*, Periostin (*Postn*), and Scleraxis (*Scx*) were analyzed by quantitative reverse transcription-polymerase chain reaction. The expression level of each gene relative to the gingival expression level, which was set to 1, is shown (n = 4 mice). Statistical analysis was performed using GraphPad Prism v9.0.2 (www.graphpad.com) (**C**) Representative images of PDL fragments on day 22 after incubation in a 6-well cell culture plate are shown. Migrating and proliferating cells (indicated by white arrowheads) could be observed around the PDL fragments. Scale bar: 500 μm. (**D**) Alizarin red S staining of murine PDL cells cultured in the normal or osteogenic medium was performed on days 8 and 16. Representative images of triplicate wells. *p < 0.05. (**E**) The quantification of Alizarin staining area performed. Each value was normalized to the value of osteogenic induction on day 16. The area analysis was performed by ImageJ software v1.53c (imagej.net).

We used micro-instruments to collect PDL from the root surface of the maxillary first molar of 6-week-old C57BL/6J mice (Supplementary Movie S1) under a stereomicroscope. Microscopic and histological analysis of PDL-scraped root surface suggested that the PDL was evenly collected (Fig. 1A). The mRNA expression levels of PDL marker genes, such as *Plap-1/Aspn*, *Postn*, and *Scx*, were compared between the collected PDL and gingiva. Higher expression was observed in the PDL (Fig. 1B), suggesting that pure PDL tissue was successfully collected. Subsequently, the explants were cultured in the FGF-2-containing medium, and the migrating and proliferating cells (Fig. 1C) were cultured as bulk murine PDL cells. The cells formed Alizarin Red-positive calcified nodules when they were cultured in osteogenic medium, indicating that the cells possessed high osteogenic capacity (Fig. 1D). Then, single cells were sorted in two 96-well plates using a cell sorter (192 wells). The single cell-derived colonies were identified and subcultured after 2 weeks, and finally, 35 clonal cell lines were established.

The clones were freeze-stocked, and osteogenic differentiation was induced by Alizarin Red S staining for 16 d to screen the cells for their osteogenic potential (Supplementary Figure S1A). Three barely stained clones were selected as the low clones (individually designated L1, L2, and L3) (Supplementary Figure S1B), and three strongly stained clones were regarded as the high clones (individually designated H1, H2, and H3) (Supplementary Figure S1C). These PDL clones were confirmed to express PDL marker genes significantly higher than gingival fibroblasts (Supplementary Figure S1D).

### Characterization of the high and low clones

**Figure 2:**
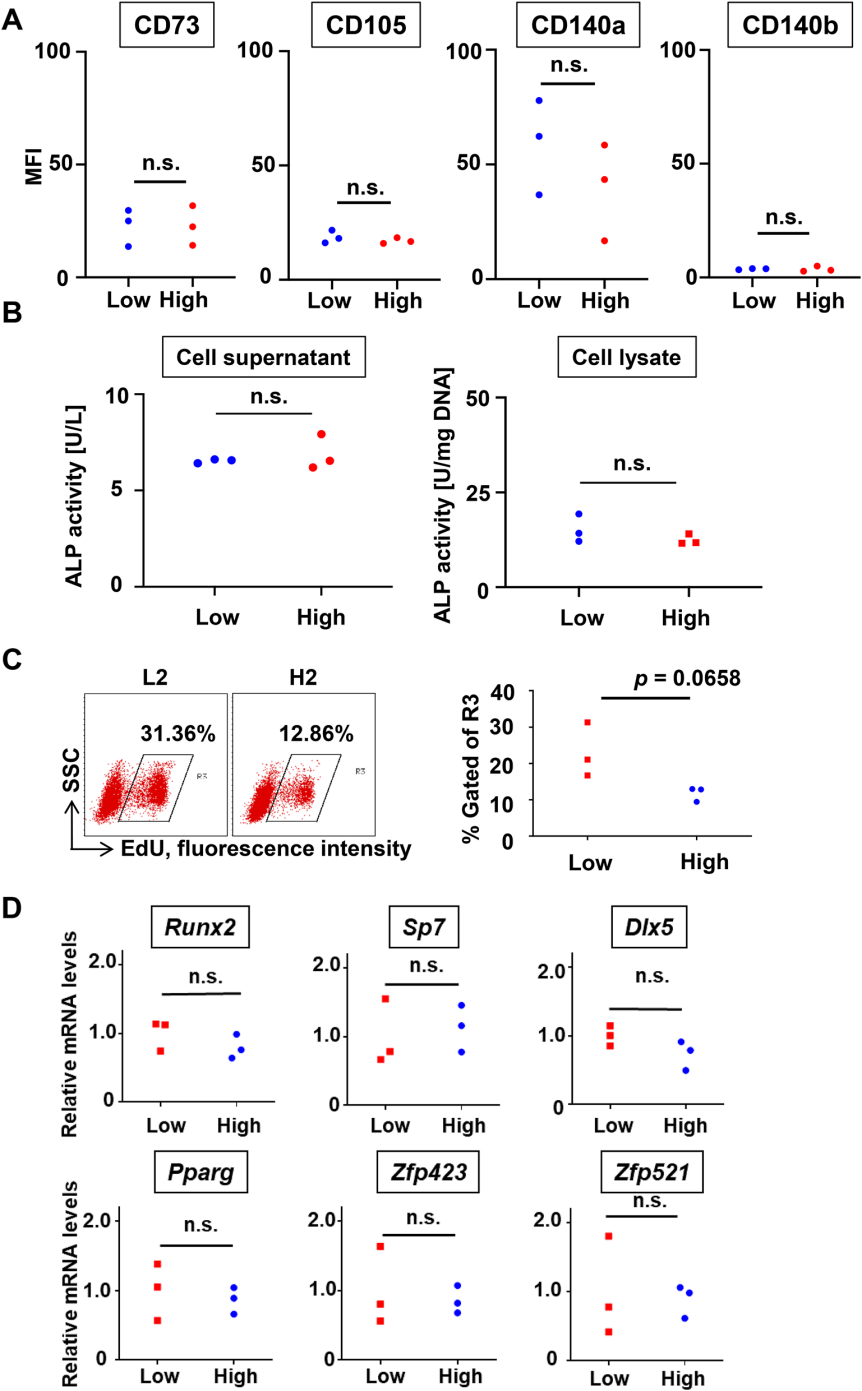
Characterization of progenitor clones with high (high clones) and low (low clones) osteogenic potential. (**A**) Flow cytometry analysis of stem/progenitor markers (CD73, CD105, CD140a, CD140b), showing mean fluorescence intensity (MFI) values, was performed. (**B**) Alkaline phosphatase activity in the cell supernatant (left) and lysate (right) was compared between the high and low clones. (**C**) The representative dot plots of in vitro EdU proliferation assay are shown (left). MFI values of all clones are shown (right, n = 3 clones). (**D**) The relative mRNA expression levels of cellular differentiation-related genes were determined by quantitative reverse transcription-polymerase chain reaction. The expression level of each gene relative to the mean value of L1-3 expression, which was set to 1, is shown. n.s., not significant. Statistical analysis was performed using GraphPad Prism v9.0.2 (www.graphpad.com).

To characterize these cell lines, we first investigated the known differentiation-related mechanisms. We compared the cell morphology, stem/progenitor cell marker expression, ALP activity, proliferation capacity, and differentiation-related gene mRNA and protein expression of the low and high clones. As a result, no difference in cell morphology between the clones was observed (Supplementary Figure S2A). For the cell surface antigen, all six clones showed high expression of CD73, CD105, and CD140a, but not CD140b (Supplementary Figure S2B), and no significant difference in MFI was observed (Fig. 2A). No significant difference in ALP activity in the cell supernatant and lysate between the clones was observed (Fig. 2B), suggesting that the enzymatic activity of ALP (an enzyme essential for osteogenesis) before osteogenic induction was comparable between these clones. With respect to the proliferative capacity, the low clones showed a higher trend, but the difference between the clones was not statistically significant (Fig. 2C). No significant difference in the osteoblast-related gene mRNA expression between the clones was observed (Fig. 2D). Concerning protein expression, all six clones stably expressed Runx2, but not its downstream protein Sp7 without osteogenic induction (Supplementary Figure S2C). Despite a clear difference in the osteogenic differentiation potential between these clones, these results showed no significant differences in their known differentiation-related mechanisms.

### RNA-seq analysis was performed to identify osteogenic progenitor-related genes

**Figure 3:**
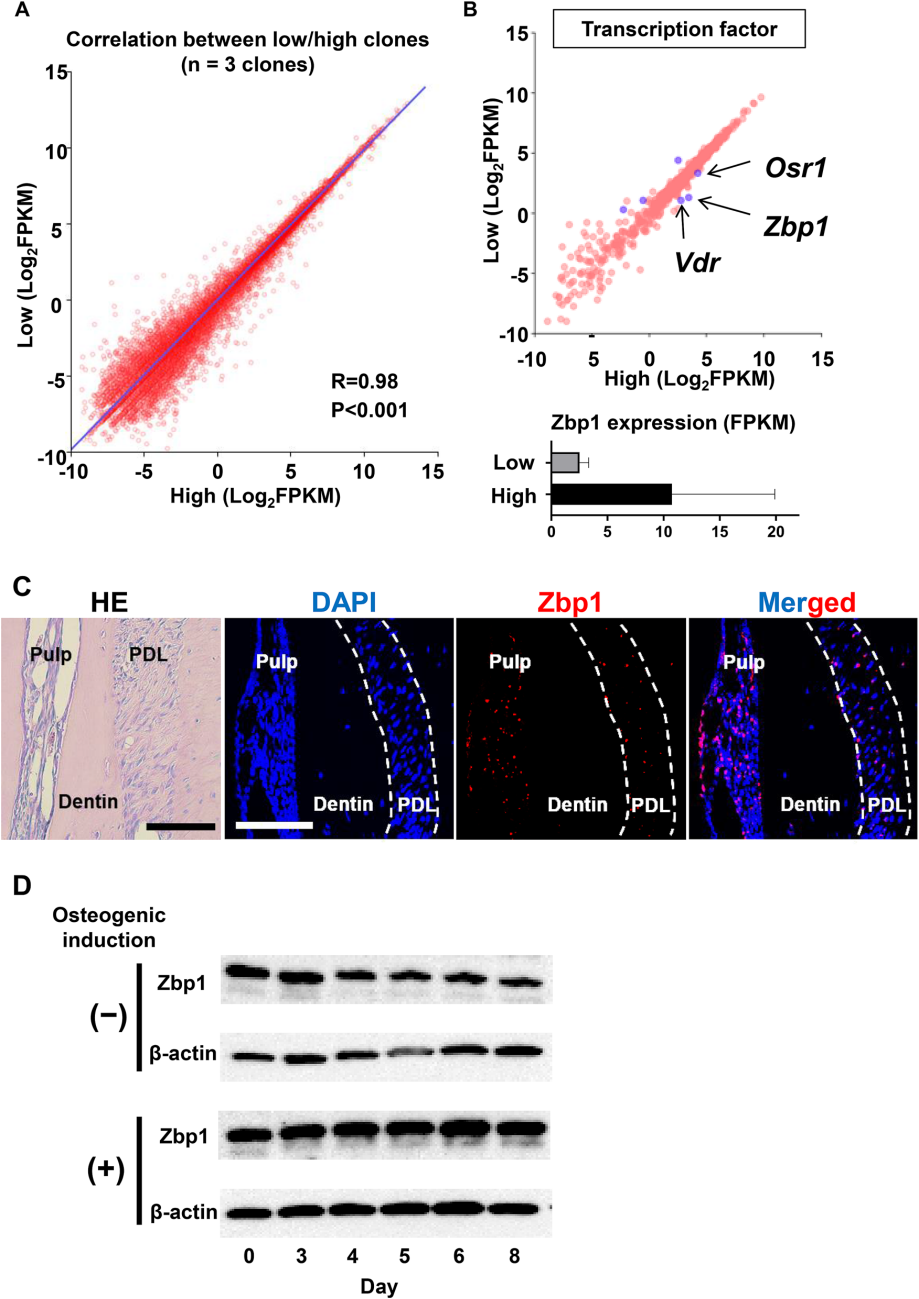
RNA-seq analysis of progenitor clones with high and low osteogenic potential. (**A**) Scatter plots of the mean values of three clones of each type calculated by RNA-seq analysis are shown. FPKM: fragments per kilobase of exon per million reads mapped. (**B**) Among the genes in A, genes encoding transcriptional regulators are shown. The differentially expressed genes (DEGs, showing > twofold statistically significant differences) are shown in purple. The Zbp1 expression (FPKM) is shown in a bar chart. (**C**) Representative images of hematoxylin and eosin staining and RNAscope in situ hybridization of adult periodontal ligament (PDL) are shown. *Zbp1* signals are shown in red. Neighboring slides were used. The PDL is shown as a white dotted line. Scale bar: 100 μm. (**D**) High clones were seeded into plates in the absence of FGF-2 and cultured with osteogenic medium on the next day. Whole cell lysate were collected at days 0, 3, 4, 5, 6, and 8 of osteogenic induction, respectively, and the representative Western blotting results for Zbp1 and β-actin are shown. The original image and quantification data are included in Supplementary Figure S6.

To identify osteogenic potential-associated molecules comprehensively, we performed RNA-seq analysis of the RNA extracted from L1-3 and H1-3. The most common gene expression patterns in both types of clones showed a significantly strong correlation (Fig. 3A). On analyzing the 1,507 transcriptional regulators (Fig. 3B), we found that three genes, including vitamin D receptor (*Vdr*), odd-skipped related-1 (*Osr1*), and *Zbp1*, were significantly expressed in the high clones and *Zbp1* showed the highest differential expression (Fig. 3B).

To investigate whether Zbp1-positive cells were present in the mouse PDL tissues, we performed RNAscope in situ hybridization of *Zbp1*. *Zbp1* mRNA expressing cells were found to be sparsely distributed throughout the PDL without aggregating near the PDL bone, cementum, or blood vessels (Figs. 3C and S3A). Zbp1-positive cells were also found in the tooth pulp, where the cells were more densely located (Figs. 3C and S3A).

Since Zbp1-positive cells were found in the PDL, Zbp1 expression was investigated by western blotting during the osteogenic differentiation of PDL cells. The results showed that Zbp1 protein expression was reduced when the cells were continuously cultured without induction but remained the same when cell differentiation was induced (Fig. 3D). The Zbp1 expressions in other undifferentiated stromal cells from bone marrow, adipose tissue, and dental pulp were also examined (Figure S3B). Interestingly, the high clone and bone marrow-derived undifferentiated stromal cells expressed Zbp1 at a higher level than the low clone and other cells. The other undifferentiated stromal cells were not single cell-derived clones and maybe heterogeneous cell populations with various Zbp1 expression levels. This possibility can be addressed by clonal analysis of these cells in future studies.

### Analysis of the effects of Zbp1-KO on the osteogenic differentiation of PDL cells

**Figure 4:**
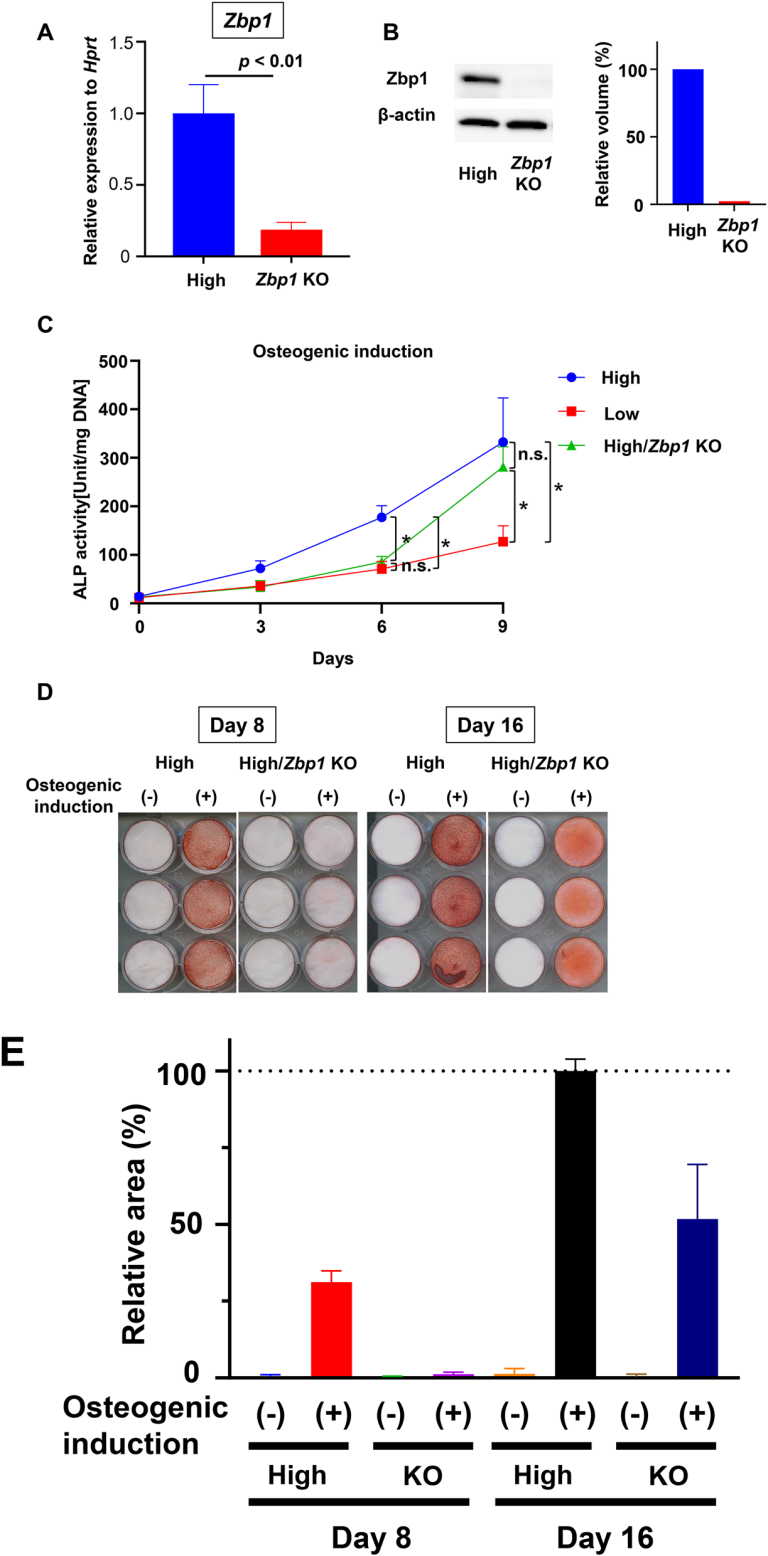
The effects of *Zbp1* knockout (KO) on the osteogenic differentiation of murine periodontal ligament (PDL) cells. (**A**) Quantitative reverse transcription-polymerase chain reaction analysis of *Zbp1* mRNA expression is shown (n = 3 wells). (**B**) Western blot analysis using anti-Zbp1 and anti-β-actin antibodies was performed. The original image is included in Supplementary Figure S6. The quantification of the band volume was performed. Each value was normalized to the value of the control sample. (**C**) Alkaline phosphatase (ALP) enzymatic activity during osteogenic differentiation was measured. High, low, and KO clones were cultured in osteogenic medium, and their ALP activity was measured on days 0, 3, 6, and 9 (n = 3 wells). The results of the analysis are shown as the mean ± standard error. (**D**) High and KO clones were cultured in osteogenic medium and stained with Alizarin Red S on days 8 and 16 of differentiation induction. The experiments were performed in triplicate (n = 3 wells) for each group. **E**. The quantification of Alizarin staining area performed. Each value was normalized to the value of osteogenic induction on day 16 of the High clone. Statistical analysis was performed using GraphPad Prism v9.0.2 (www.graphpad.com). The area analysis was performed by ImageJ software v1.53c (imagej.net).

Next, to analyze the Zbp1 function during osteogenic differentiation, we generated a *Zbp1*-KO cell clone (High/*Zbp1*-KO) by knocking out the *Zbp1* gene in the high clone by editing the CRISPR/Cas9 genome that targeted exon 3 (Supplementary Figure S4A). We confirmed successful genome editing by Sanger sequencing (Supplementary Figure S4B), qRT-PCR (Fig. 4A), and western blotting (Fig. 4B). The KO, high, and low clones were incubated in osteogenic medium for 9 d, and their ALP activity was quantiified every 3 d. On day 6, the KO clone showed significantly lower ALP activity, which was as low as that of the low clone, than the high clone. However, by day 9, the ALP activity of the KO clone returned to as high as that of the high clone (Fig. 4C). When the high and KO clones were cultured in osteogenic medium, the KO clone showed lesser calcified nodule formation on day 8; however, on day 16, the KO clone was almost similarly stained as the high clone (Fig. 4D,E). These results suggested that Zbp1 might promote the differentiation of Zbp1-positive cells into osteoblasts, especially in the early stages.

Furthermore, we attempted to generate several KO clones (High/Zbp1 KO2). Sanger sequencing analysis revealed these clones had a variety of indel mutations (Supplementary Figures S5A–D). Western blotting revealed that KO2 clones #1, #3, #5, #6, and #7 did not express the Zbp1 protein (Supplementary Figure S5E). When the KO2 and high clones were cultured in osteogenic medium for 8 d, KO2 clones #1, #3, #5, and #7 showed little staining and KO2 clone #6 was similarly stained as the high clone (Supplementary Figure S5F). The presence or absence of Zbp1 expression mostly coincided with the osteogenic capacity of the Zbp1-positive cells.

### Analysis of the effects of Zbp1 overexpression on osteogenic differentiation

**Figure 5:**
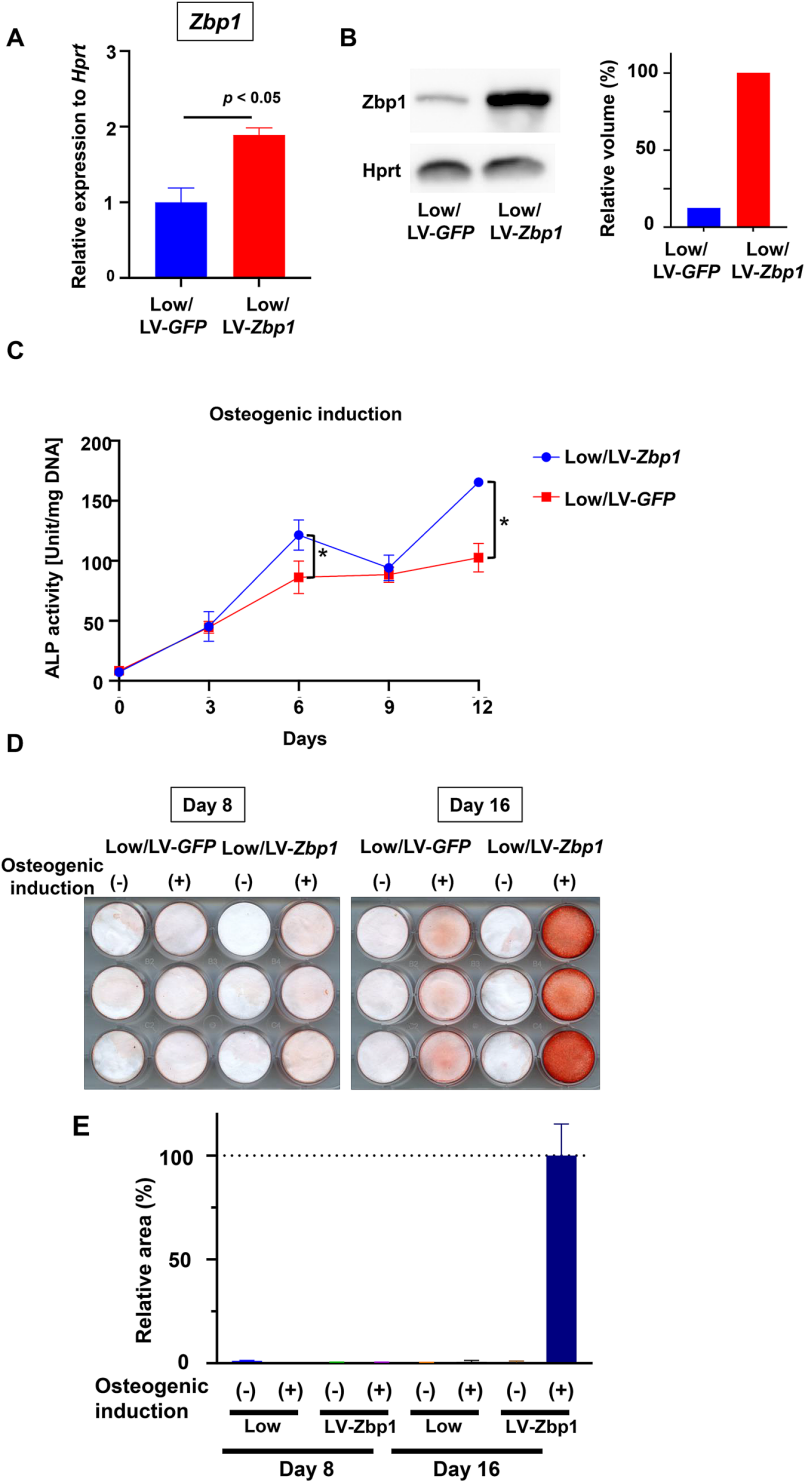
The effects of *Zbp1* overexpression on the osteogenic differentiation of murine periodontal ligament (PDL) cells. (**A**) *Zbp1* mRNA expression was analyzed by quantitative reverse transcription-polymerase chain reaction (n = 3 wells). (**B**) Western blotting using anti-Zbp1 and anti-Hprt antibodies is shown. The original image is included in Supplementary Figure S6. The quantification of the band volume was performed. Each value was normalized to the value of the control sample. (**C**) Low/LV-Zbp1 and Low/LV-GFP were cultured in osteogenic medium, and their ALP activity was measured on days 0, 3, 6, 9, and 12 of differentiation induction (n = 3 wells). The results of the analysis are shown as the mean ± standard error. (**D**) Low/LV-Zbp1 and Low/LV-GFP were cultured in osteogenic medium, and differentiation was induced on days 8 and 16. The experiments were performed in triplicate (n = 3 wells) for each group. (**E**) The quantification of Alizarin staining area performed. Each value was normalized to the value of osteogenic induction on day 16 of the Low/LV-Zbp1. Statistical analysis was performed using GraphPad Prism v9.0.2 (www.graphpad.com). The area analysis was performed by ImageJ software v1.53c (imagej.net).

To analyze the effects of Zbp1 on osteogenic differentiation further, we generated a Zbp1-expressing low clone using a lentivirus. qRT-PCR (Fig. 5A) and western blotting (Fig. 5B) results confirmed that Zbp1 was strongly expressed in the transduced cells. The Zbp1-expressing cells were then cultured in osteogenic medium, and they showed significantly higher ALP activity than the low clone (Fig. 5C). Neither the Zbp1-overexpressing cells nor the control cells formed calcified nodules by day 8, but calcified nodule formation was enhanced in the Zbp1-overexpressing cells by day 16 (Fig. 5D,E). This functional analysis of Zbp1 suggested that Zbp1 might promote osteogenic differentiation.

### Analysis of periodontal tissue of *Zbp1* KO mice

**Figure 6:**
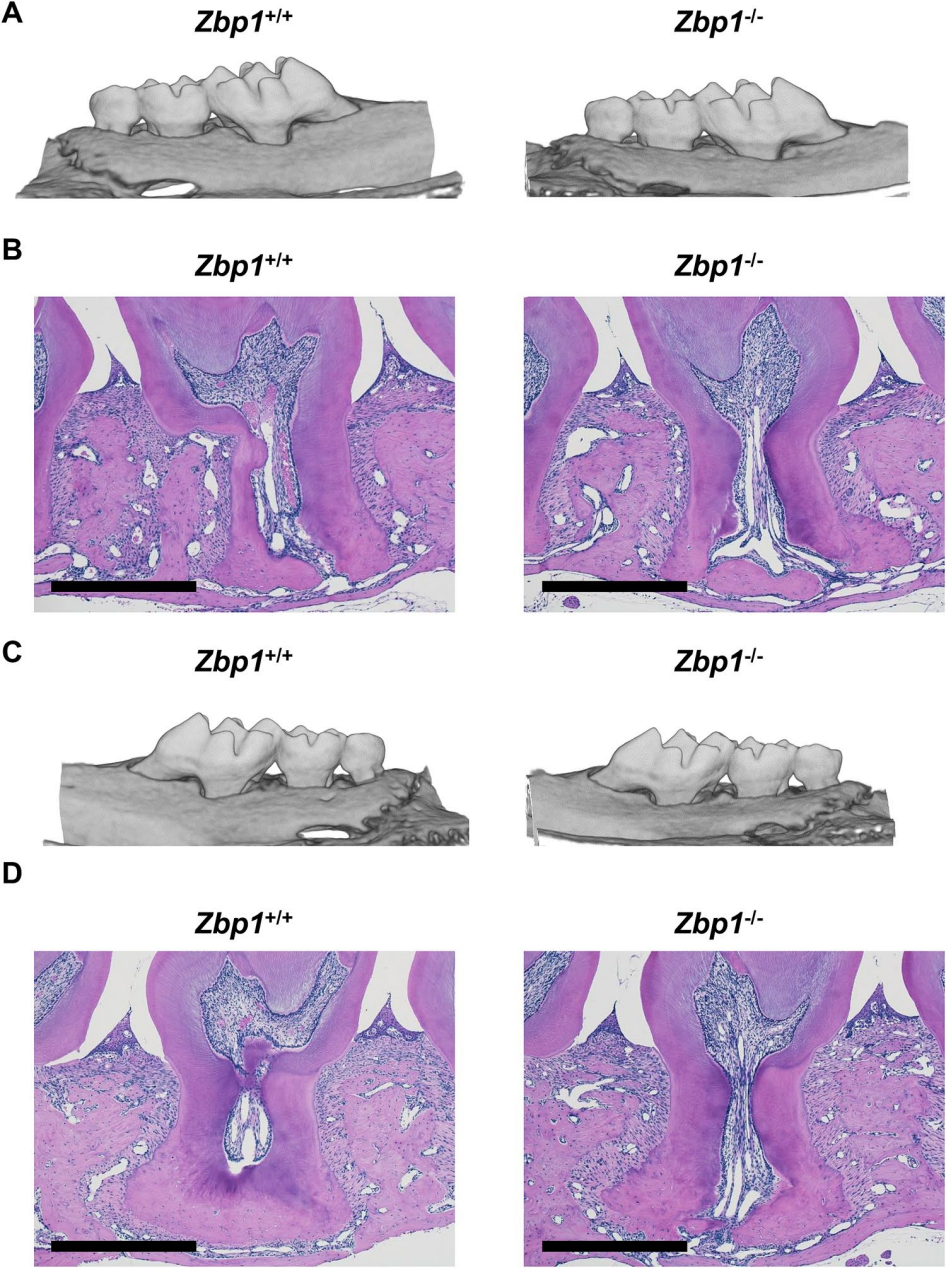
Analysis of *Zbp1* KO mice. (**A**) The representative micro-CT images of intact maxillae of 10-week-old Zbp1 KO and WT mice. (**B**) The representative HE staining images of intact maxillary left second molar of 10-week-old Zbp1 KO and WT mice. (**C**) The representative micro-CT images of repaired maxillae of 10-week-old Zbp1 KO and WT mice. (**D**) The representative HE staining images of repaired maxillary left second molar of 10-week-old Zbp1 KO and WT mice. Scale bar: 500 μm.

*Zbp1* KO mice are viable and fertile with no gross abnormalities^17,18^. We evaluated their alveolar bone morphology and periodontal tissue histology by micro-CT and HE staining, respectively. We did not see a clear difference in bone and teeth morphology as well as periodontal tissue histology (Fig. 6A,B). To analyze how the Zbp1 functions when periodontal tissue is repaired, we utilized a ligature induced periodontitis model where the periodontal tissue is damaged upon a ligature placement but repaired after the removal of the ligature. We did not see a clear delay in the repair in these settings (Fig. 6C,D).

## Discussion

During periodontal tissue development, dental follicle cells derived from the cranial neural crest differentiate into stem/progenitor cells of periodontal tissues, including the PDL^19^. However, how adult stem/progenitor cells contribute to periodontal tissue homeostasis and repair remains to be elucidated. In this study, we established progenitor cell clones with high and low osteogenic potential from adult murine PDL cells by applying a novel protocol and comprehensively analyzed their gene expression to identify the genes associated with their osteogenic differentiation. By applying a similar screening strategy using 42 Swiss 3T3 fibroblast subclones, Zfp423 was found to be a novel adipocyte differentiation regulator and adipocyte precursor cell marker^20^. In another study on osteoblast lineage, 15 subclones were established from the MC3T3-E1 osteoblast precursor^21^, and their osteogenic potential was analyzed in detail^22^. In addition, using a microarray approach of single CFU-F derived cells from human PDL, dental pulp, and bone marrow, Menicanin et al. found a common gene profile in high growth/multi-potential cells when compared with low growth cells^23^. However, a molecule that facilitates osteogenesis has not yet been identified. In this study, we identified Zbp1 as a promoter of the osteogenic differentiation of PDL cells.

To obtain only murine PDL tissue and no other contaminant, we removed the gingiva and extracted the teeth before scraping the PDL, according to our protocol. The migrating and proliferating cells from the explants were used to isolate FGF-2-responsive cells, which are thought to be the stem/progenitor cells. Further improvement of the protocol will enable us to obtain whole single cells from the PDL for single-cell omics, which can be feasibly applied for cells from developmental periodontal tissues^19^.

Despite the apparent difference in the osteogenic potential between the high and low clones, these clones shared similar cell morphology, surface antigen composition, baseline ALP activity, proliferation capacity, and known osteoblast-related molecular expression, and no significant difference in these known differentiation-related mechanisms between these clones was observed (Fig. 2). These results suggested that the difference in the osteogenic potential of PDL cells might be related to unknown (not-yet-reported) molecules. Accordingly, to identify novel differentiation regulatory molecules comprehensively, RNA-seq analysis was performed using the RNA extracted from three clones of each type. As a result, 131 differentially expressed genes (DEGs) that showed a statistically significant difference ($\ge$ twofold) in expression were identified. In this study, we first analyzed 1,507 mouse transcription factors registered in the TcoF-DB transcription factor database^24^ as molecular candidates of osteogenic differentiation regulation and narrowed down 6 genes (Fig. 3). The three DEGs upregulated in the high clones were *Zbp1*, *Vdr*, and *Osr1*. Vdr binds to active vitamin D and is translocated to the nucleus, promoting the activation of various genes^25^. The expression of many proteins secreted by osteoblasts is enhanced by vitamin D in not only progenitor cells but also osteoblasts and osteocytes, and the various effects of this expression have been reported^26,27^. Osr1 is believed to be a progenitor cell marker in the heart and kidney^28,29^. It has been reported that Osr1 is involved in the induction of osteoblast differentiation^30^ and activated vitamin D induces its expression^31^.

Zbp1 was discovered to be a protein that binds to Z-DNA, a type of left-handed double-helical DNA structure, and has been reported to be highly expressed in human and mouse lymph nodes and spleen^32,33^. Zbp1 acts as a sensor to detect viral DNA that has entered the cytoplasm via its DNA-binding domain^34^ and induces cell death by cooperating with the Receptor Interacting Protein Kinase (RIPK), a protein believed to be responsible for the elimination of virus-infected cells. Excessive Zbp1 activation during mouse development has been reported to cause epithelial cell death in a RIPK-dependent manner^35,36^. However, Zbp1 expression and localization in periodontal tissues and its function in osteogenesis had not been studied until recently.

Zbp1-positive cells were sparsely scattered in the PDL without aggregating near the bone, cementum, or blood vessels (Figs. 3C and S3A). A number of Zbp1-positive cells were also found in dental pulp, suggesting that they may also be the hard tissue-forming precursor cells (Figs. 3C and S3A). Since apical papilla have a highly clonogenic stem cell population^37^, a similar Zbp1 distribution pattern in both the middle and apical area of PDL may suggest Zbp1-positive osteogenic progenitors are diffrerent from stem cells in apical papilla. The PDL homeostasis may be maintained by both Zbp1 expressing progenitors and other stem cell population. Studying the fate of Zbp1 expressing cells using a lineage tracing approach will be interesting. Zbp1 protein expression was decreased when cells were cultured in normal medium, but was maintained at a high level when cells were cultured in osteogenic medium (Fig. 3D), suggesting the impotance of FGF-2 to maintain the Zbp1 expression and the possible involvement of Zbp1 in osteogenic differentiation. These expression data led us to conduct experiments on Zbp1 loss-of-function and gain-of-function during osteogenic differentiation.

The loss-of-function experiment was performed by generating multiple *Zbp1* KO clones. The absence of Zbp1 expression correlated with a delay in calcified nodule formation and decreased ALP activity during early differentiation, suggesting that Zbp1 was a regulatory factor that promoted the early stages of differentiation. The gain-of-function experiments also suggested that Zbp1 was involved in osteogenic differentiation. We also analyzed adult *Zbp1* KO mice^17^ and found that they, compared with the *Zbp1* wild type mice, did not show a clear phenotype of periodontal tissues (Fig. 6). Interestingly, a very recent study also showed that Zbp1 promoted osteogenic differentiation via a positive feedback loop of Wnt/catenin signaling^38^. We concluded that even if Zbp1 was not necessary for cellular differentiation, it possibly fine-tunes the process in vivo together with other factors such as *Vdr1* and *Osr1* found in this study. The possible redundant roles of these transcription factors will need to be addressed in the future.

In summary, we established and compared PDL progenitor clones with high and low osteogenic potential and found that Zbp1 promoted osteogenic differentiation of PDL progenitor cells. Zbp1 labeled cells with high osteogenic potential. Further studies must be conducted to clarify the upstream factors of Zbp1, thereby facilitating a better understanding of periodontal tissue homeostasis and repair and development of novel therapeutic interventions.

## Materials and methods

### Animals

All animal experiments were approved by the Institutional Animal Care and Use Committee of Osaka University Graduate School of Dentistry and complied with the guidelines for the care and use of laboratory animals at Osaka University (Approval No. 31-001-0). C57BL/6J mice were purchased from Japan SLC. *Zbp1* KO mice were obtained from Prof. Shizuo Akira through Laboratory Animal Resource Bank at NIBIOHN (Resource#: nbio155). PCR genotyping is performed with primers shown in Supplementary Table S1. Mice were provided sterile food and water under specific-pathogen-free conditions. All animal experiments complied with the ARRIVE guidelines.

### Isolation and cell culture of murine PDL cells

Upon euthanization, the maxillae of C57BL/6J mice were separated, and molars were extracted under a stereomicroscope. PDL was scraped from the surface of the mesial root of the first molar using micro-instruments, including a micro-curette (0.5 mm; Fine Science Tools, CA, USA). The scraped tissue was placed in a 6-well plate under a coverslip to prevent the tissue from floating and cultured in cell culture medium (α-Modification of Eagle’s Medium; FUJIFILM Wako Pure Chemical, Japan) containing 10% fetal bovine serum (Life Technologies, CA, USA), kanamycin (60 µg/mL; FUJIFILM Wako Pure Chemical), and FGF-2 (100 ng/mL; Kaken Pharmaceutical, Japan). The medium was incubated at 37℃ in 95% air and 5% CO_2_. The cells that migrated and proliferated from the tissue were subcultured as murine PDL cells. Murine gingival fibroblasts (MGF) were established in this study. The dissected palatal gingiva of C57BL/6J mice was minced and placed in a 6-well plate with MF-start primary culture medium (Toyobo, Japan). The migrated cells from the tissue were subcultured with MF-medium (Toyobo) as MGF. Murine dental pulp-derived stromal cells (mDPSC) were also established from pulp explant of maxillary molars with the same protocol with MGF. Murine bone marrow and adipose tissue-derived undifferentiated stromal cells (mBMSC and mADSC) were purchased from Cyagen, USA.

### Osteogenic differentiation culture

To induce osteogenic differentiation, confluent cells were cultured in the osteogenic medium [cell culture medium supplemented with l-ascorbic acid phosphate magnesium salt n-hydrate (50 µg/mL) and 10 mM glycerol 2-phosphate disodium salt n-hydrate (FUJIFILM Wako Pure Chemical)], and the medium was replaced with fresh medium every 3 d. Alizarin Red S staining was performed as previously described^39^. The area analysis was performed by ImageJ software v1.53c^40^ (imagej.net).

### Cloning and screening of murine PDL cells

Single-cell sorting was performed using the SH800Z Cell Sorter (Sony Biotechnology, CA, USA) with the 100-μm microfluidic sorting chips set on a single-cell mode. Cells were sorted into 96-well plates containing the cell culture medium (200 µL) with FGF-2. After 14 d, no multiple colonies per well were found, and a single cell-derived colony was passaged in 24-well plates, 6-well plates, and finally 10-cm dishes.

### Bulk RNA-seq

RNA-seq libraries were prepared from the total RNA isolated from the cultured osteogenic and non-osteogenic clones (n = 3 each), sequenced with HiSeq2500 (Illumina) in 2 × 101 bp paired-end sequencing, and analyzed by Hokkaido System Science. The data were further visualized using the Python visualization library.

### Generation of Z-DNA binding protein-1 (Zbp1)-knockout (KO) subclones

We performed *Zbp1* loss-of-function in the high clones using CRISPR/Cas9 as previously described^39^. First, we designed the guide RNA that targeted exon 3. Cas9 protein/guide RNA complex (Fig S4) was transfected using the Neon Transfection System with 10-µL tips. One day after transfection, cells were sorted, and subclones were established as described above. The genomic DNA was obtained when the passaged cells and the positive clones were screened by MultiNA (Shimadzu, Kyoto, Japan) capillary electrophoresis of polymerase chain reaction (PCR) amplicons (Supplementary Table S1) for the targeted genome region. Subsequently, sequencing analysis was performed with primers, which are shown in Supplementary Table S1. Subsequently, PCR product was mixed with pBluescript KS + vector with NEBuilder HiFi DNA Assembly Master Mix (New England Biolabs, MA, USA). The mixture was then transformed into NEB5α competent cells (New England Biolabs). The purified DNA from each colony was subject to sequencing analysis. mRNA and protein expression levels of the KO clones were analyzed by quantitative reverse transcription-PCR (qRT-PCR) and western blotting, respectively.

### Lentivirus-mediated Zbp1 overexpression in low clones

Low clones were transduced with Zbp1-expressing lentiviral particles (pLV[Exp]-Puro-CMV > mZbp, packaged by VectorBuilder) or control green fluorescent protein lentiviral particles at a multiplicity of infection of 10. The infected cells were selected and maintained in puromycin. mRNA and protein expression levels of the *Zbp1*-overexpressing cells were analyzed by qRT-PCR and western blotting, respectively.

### qRT-PCR

Total RNA was extracted from cells with Purelink RNA Mini Kit (Thermo Fisher Scientific), and cDNA was synthesized with SuperScript III reverse transcriptase (Thermo Fisher Scientific) according to the manufacturer’s instructions. PCR was performed with StepOnePlus Real-time PCR System (Applied Biosystems) and SYBR Green PCR Master Mix (Applied Biosystems). The expression level of target genes was normalized to a housekeeping gene (*Hprt1* or *B2m*) and control group. The primer pairs used in this study were shown in Supplementary Table S1.

### Western blot

Cells were washed with PBS and lysed using RIPA lysis buffer containing protease inhibitor cocktail, 10 mM NaF, and 1 mM Na_3_VO_4_. Whole-cell lysate was separated by SDS-PAGE and transferred on PVDF membrane with Transblot Turbo Transfer System (Bio-Rad). The membrane was blocked with 5% skim milk in TBS-T buffer for 30 min at RT and followed by incubation with the primary antibodies (Supplementary Table S2) in CanGetSignal solution (TOYOBO) for 2 h at RT. Following TBS-T washes, the membranes were incubated with HRP conjugated secondary antibody (Supplementary Table S2) for 30 min at RT. Then membrane was incubated with SuperSignal West Dura Extended Duration Substrate (Thermo Fisher Scientific) for 5 min and chemiluminescence signal was detected by ImageQuant LAS 4000 (GE Healthcare). The band volume analysis was performed by Image Lab v6.0.1 (Bio-Rad) according to the manufacturer’s instructions, and the band volume relative to the loading control was further normalized to the value of the control sample. The detailed descriptions for the analysis are shown in Supplementary Table S3.

### Flow cytometry analysis

The cells were harvested with Trypsin–EDTA and resuspended in FACS buffer (PBS containing 5% FBS). The cell pellet was then stained for 30 min on ice with fluorescent protein conjugated antibodies (Supplementary Table S2). Flow cytometry analysis was performed on FACS Calibur (BD) flow cytometer, and the mean fluorescent intensity was calculated by CellQuest software (BD).

### Alkaline phosphatase (ALP) enzymatic activity assay

After washing PBS, the cells were homogenized in sterile distilled water with Handy Sonic model UR-20P (Tomy). The lysate was mixed with 0.5 M Tris–HCl buffer (pH 9.0), 0.5 mM p-nitrophenyl phosphate (pNPP) and 0.5 mM MgCl_2_. The samples were incubated for 30 min, and the reaction was stopped by the addition of 0.25 mL of 1 N NaOH. The absorbance of the solution at a wavelength of 405 nm was measured with a Multiskan FC Microplate Reader (Thermo Fisher Scientific) to determine the amount of hydrolyzed pNPP. The value of activities was normalized by DNA content for lysate or supernatant volume for culture supernatant, respectively.

### RNAscope in situ hybridization

Maxilla of 6-week-old C57BL/6J mice was dissected, fixed with 10% Neutral buffered formalin (Wako), decalcified with Morse solution (Wako), embedded in paraffin, and sectioned at 7 μm. The detection of Zbp1 signals was performed by the RNAscope system (ADC) according to the manufacturer’s procedure. The counterstained sections were imaged with Leica SP8 confocal microscope.

### In vitro EdU proliferation assay

The cells were incubated with 20 µM EdU (Wako) for 20 min, and EdU positive cells were detected using the FACS Calibur after performing Click chemistry reaction according to the manufacturer’s protocol (Life Technologies).

### Ligature-induced periodontitis model

To induce periodontitis, a 5–0 silk ligature was tied around the maxillary left second molar of 8-week-old *Zbp1* KO and WT mice as described previously^41,42^. After 7 days, the ligature was removed. The recovery of the tissue was analyzed at day 7 after the removal. The contralateral molar tooth in each mouse was left unligated as intact periodontal tissue.

### Micro-computed tomography scan analysis

The dissected maxillae tissue were fixed with 4% PFA overnight and scanned in R_mCT2 3D micro X-ray CT system (Rigaku, Japan) at a voxel size of 5 mm^3^ for 3 min (90 kV and 200 μA). Maxillary tissues were reconstructed and analyzed via Amira software (Thermo Fisher Scientific).

### Statistical analysis

Data are presented as mean ± standard deviation (SD) in Figs. 1E, 3B, 4A,C,E, 5A,C,E, S1D, S3B, S5G or mean ± standard error (SEM) in Fig. 1B otherwise raw data values were plotted. Statistical analysis was performed using GraphPad Prism v9.0.2 (GraphPad Software, USA, www.graphpad.com) and detailed descriptions for the analysis are shown in Supplementary Table S3. Statistical significance was assumed at *p* < 0.05.

## Supplementary Information

Supplementary Information 1.

Supplementary Video 1.

## Data Availability

All data needed to evaluate the conclusions in the paper are present in the paper and/or the Supplementary Information. Additional data related to this paper may be requested from the authors.
