# Supplementary material for: Zbp1-positive cells are osteogenic progenitors in periodontal ligament
Source: Sci Rep. 2021 Apr 6;11:7514. doi: 10.1038/s41598-021-87016-1 (PMC8024286; doi:10.1038/s41598-021-87016-1)
Supplement: Supplementary file 1 — Supplementary Information 1. [file 41598_2021_87016_MOESM1_ESM.pdf]

Zbp1-positive cells are osteogenic progenitors in periodontal ligament

Tsugumi Ueda, Tomoaki Iwayama*, Kiwako Tomita, Shuji Matsumoto, Mizuho Iwashita, Phan Bhongsatiern, Hiromi Sakashita, Chiharu Fujihara, Masahide Takedachi, and Shinya Murakami*

Department of Periodontology, Osaka University Graduate School of Dentistry, Suita, Osaka, Japan

Supplementary Fig S1

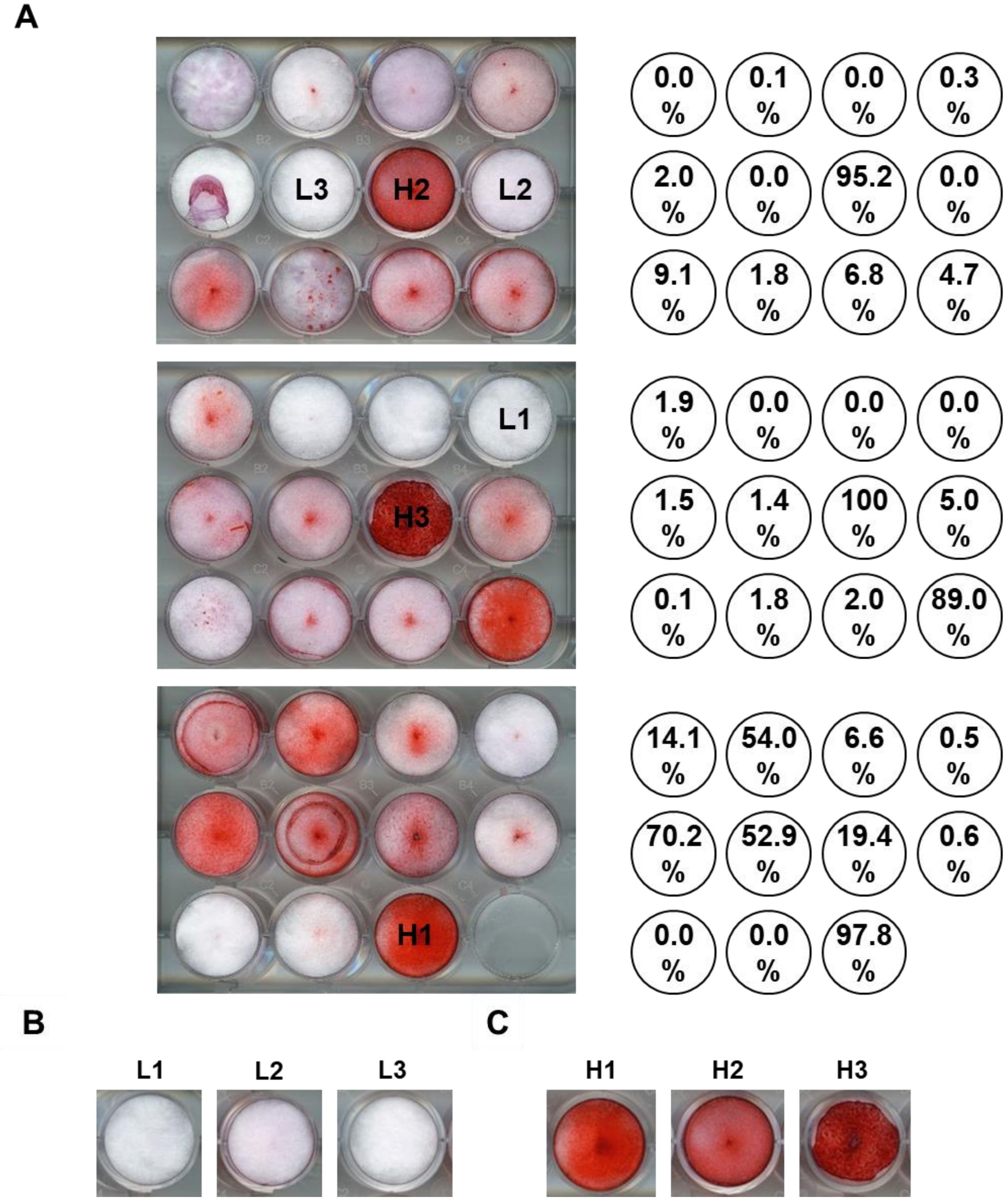

Supplementary Fig S1 cont'd

D

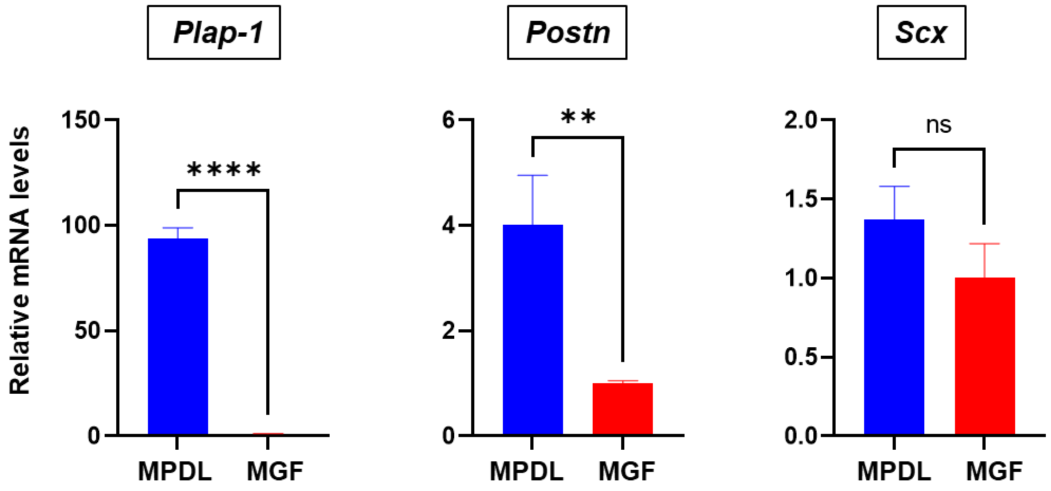

Supplementary Fig S1: Establishment of mouse PDL-derived cell clones.

A. All 35 established clones were cultured in osteogenic medium and stained with Alizarin Red S on day 16 to induce differentiation. Quantification of Alizarin Red S staining area performed. Each value was normalized to the value of the H3 clone. Of these clones, three were selected as low clones (B: only clones selected from A), and three were selected as high clones (C: only clones selected from A), which were stained red. D. The mRNA expression levels of PDL markers *Plap-1/Aspn*, *Periostin* (*Postn*), and *Scleraxis* (*Scx*) were analyzed by quantitative reverse transcription-polymerase chain reaction (n=3 wells). **p <0.01, ***p <0.001, ns: not significant, MGFs: mouse gingival fibroblasts. Statistical analysis was performed using GraphPad Prism v9.0.2 (www.graphpad.com). The area analysis was performed by ImageJ software v1.53c (imagej.net).

Supplementary Fig S2

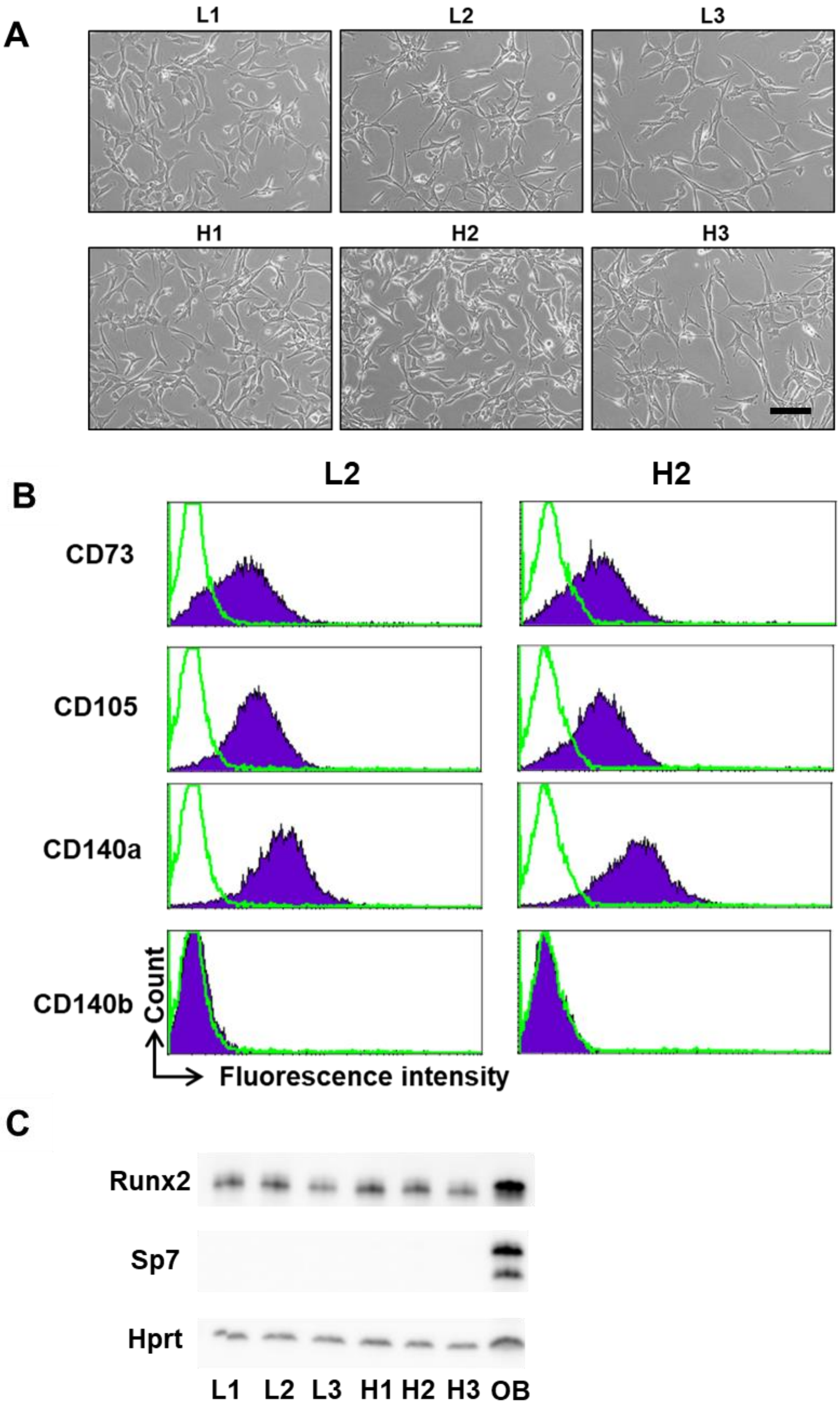

OB : osteoblastic cells line, KUSA-A1

Supplementary Fig S2: Characterization of High and Low osteogenic progenitors

A. Representative phase-contrast images of the Low (L1, L2, L3) and High (H1, H2, H3) clones, respectively, are shown. Scale bar: 200 μm. B. Representative histogram plot of surface marker expression of the low (L2) and high (H2) clones. MFI data is shown in Fig. 2A. C. The expression of Runx2 and Sp7 was examined by the Western blot analysis. Whole-cell lysate from an osteoblastic cell line, KUSA-A1 was used as a positive control. The original image and densitometry quantification data are included in Supplementary Figure S6.

Supplementary Fig S3

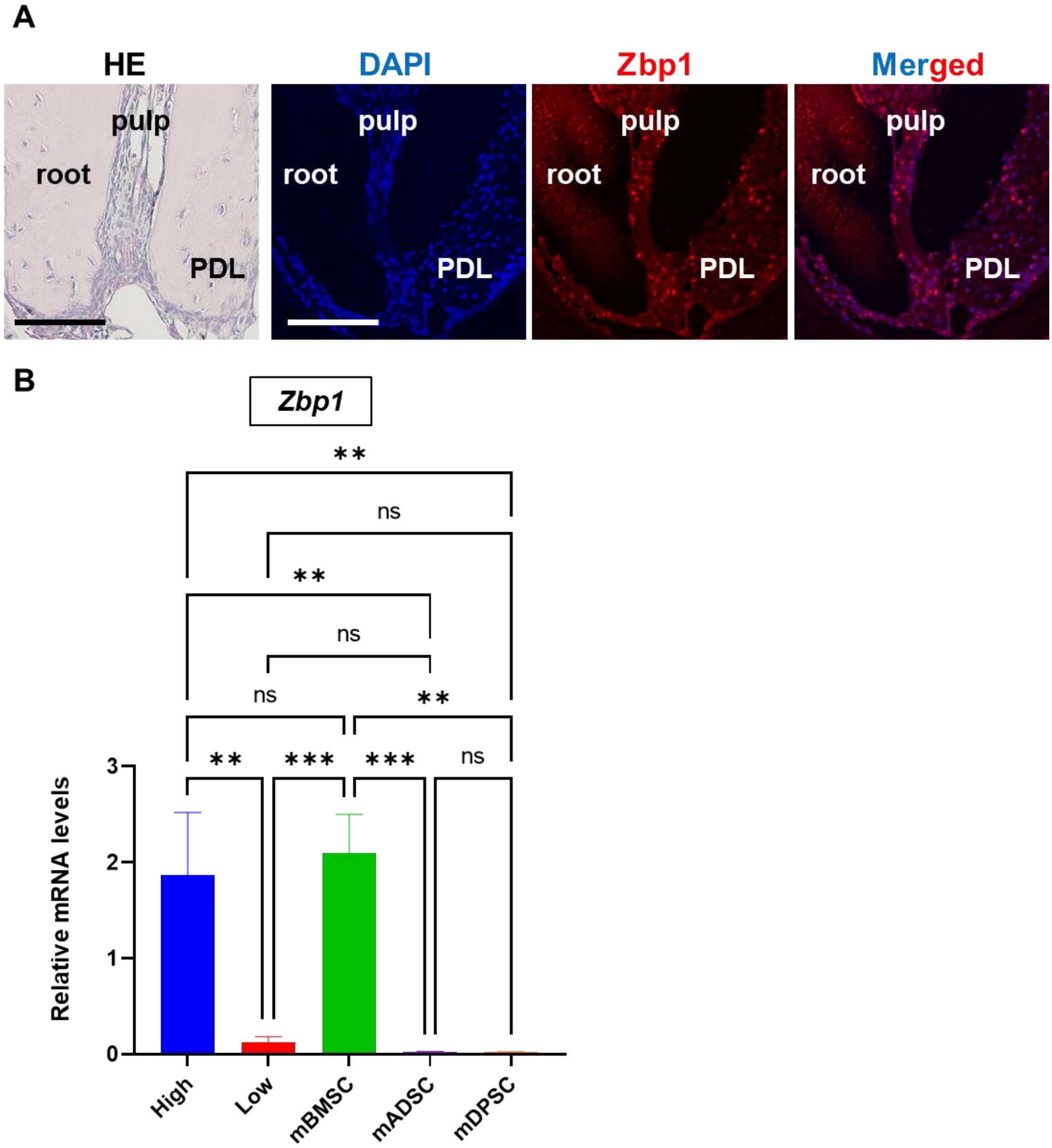

Supplementary Fig S3: *Zbp1* expessions in periodontal tissue and cultured undifferentiated cells

A. Representative images of hematoxylin and eosin staining and RNAscope *in situ* hybridization of apical area of adult M2 tooth are shown. Zbp1 signals are shown in red. Scale bar: 100 µm. B. The schematic diagram of the *Zbp1* gene and target site of CRISPR-Cas9 is shown. B. The mRNA expression levels of *Zbp1* were analyzed by quantitative reverse transcription-polymerase chain reaction (n=3 wells). **p <0.01, ***p<0.001. ns: not significant. mBMSC: murine bone marrow-derived undifferentiated stromal cell. mADSC: murine adipose tissue-derived undifferentiated stromal cell. mDPSC: murine dental pulp derived undifferentiated stromal cell. Statistical analysis was performed using GraphPad Prism v9.0.2 (www.graphpad.com).

Supplementary Fig S4

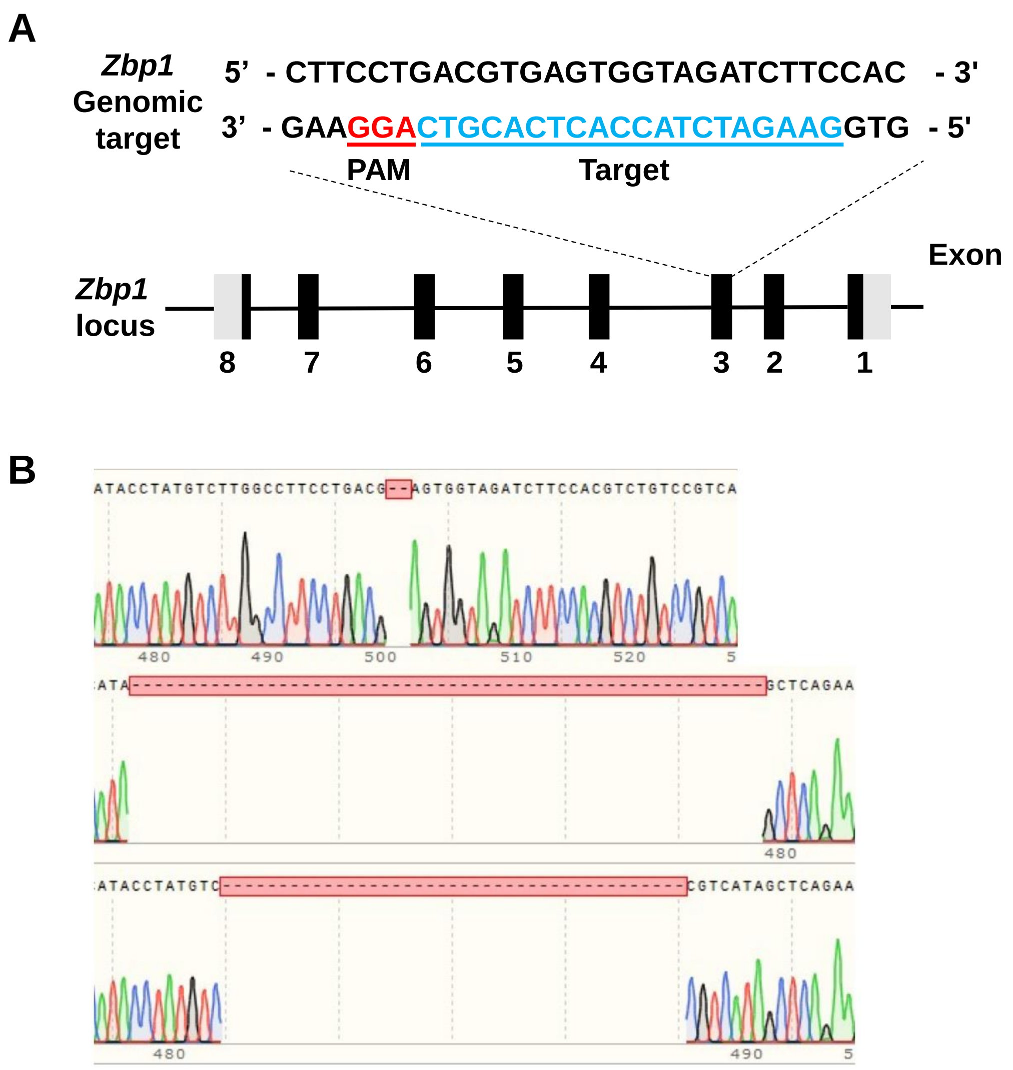

Supplementary Fig S4: Genomic characterization of ***Zbp1*** KO cells

A. The schematic diagram of the *Zbp1* gene and target site of CRISPR-Cas9 is shown. B.

The results of the sequencing analysis of the High/Zbp1 KO clone.

Supplementary Fig S5

A

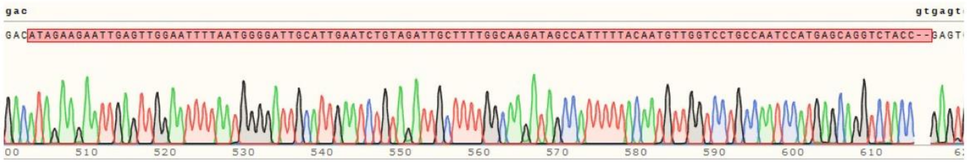

B

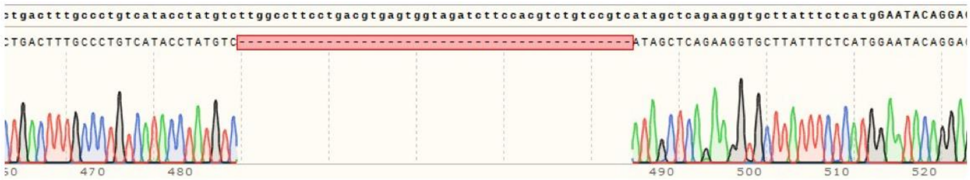

C

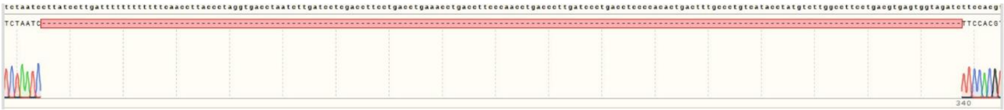

D

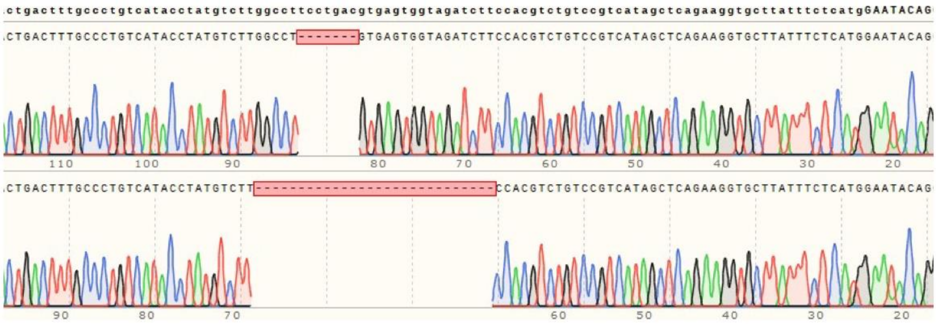

E

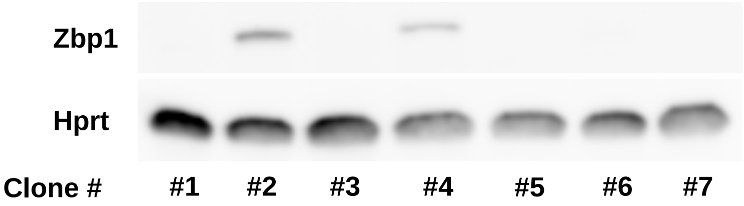

F

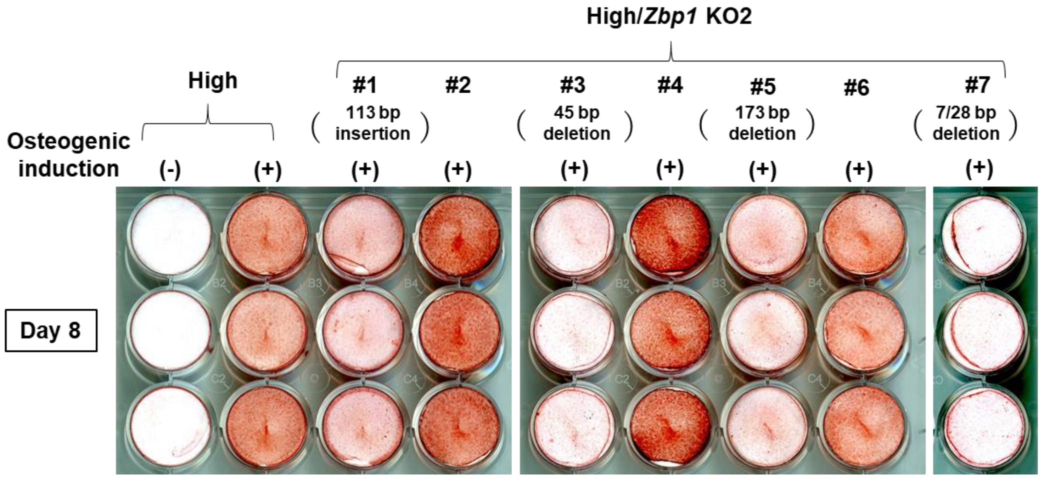

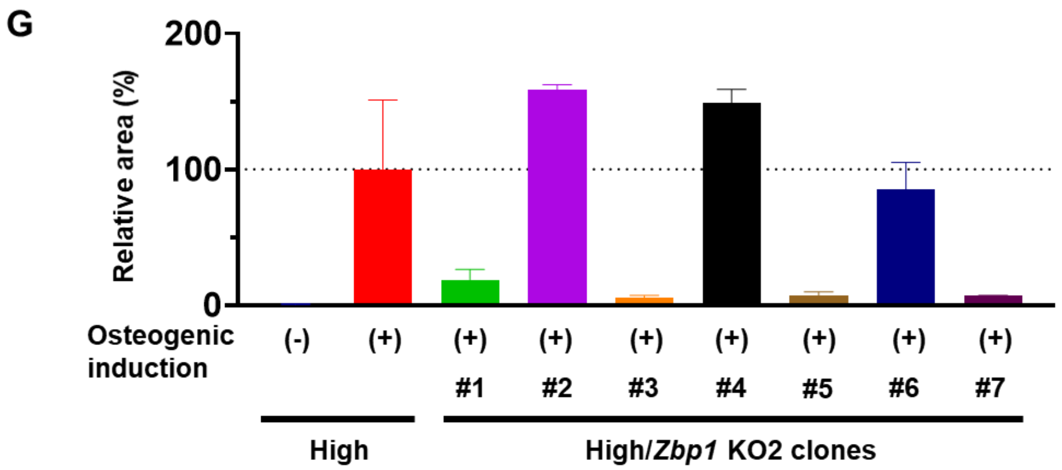
G
200
100
0
Relative area (%)
Osteogenic induction
(-)
(+)
(+)
(+)
(+)
(+)
(+)
(+)
(+)
#1
#2
#3
#4
#5
#6
#7
High
High/*Zbp1* KO2 clones

Supplementary Fig S5: characterization of other *Zbp1* KO subclones.

A-D. The results of Sanger sequencing analysis of *Zbp1* KO2 #1, #3, #5, and #7 are shown, respectively. E. Zbp1 expression of the 7 KO candidate clones was analyzed by Western blot using anti-Zbp1 and anti-Hprt antibodies. The original image and quantification data are included in Supplementary Fig S6. F. 7 KO candidate clones as well as a high clone were cultured in osteogenic medium and stained with Alizarin Red S at 8 days of differentiation induction. The experiments were performed in triplicate wells for each group. G. Quantification of Alizarin Red S staining area performed. Each value was normalized to the value of osteogenic induction of the High clone. The area analysis was performed by ImageJ software v1.53c (imagej.net).

Supplementary Fig S6: Immunoblots performed in this study.

Fig. 3D

anti-Zbp1

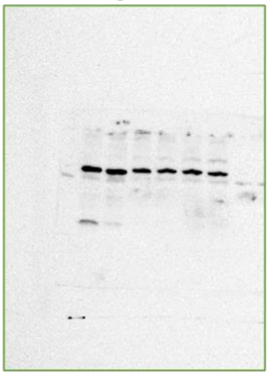

anti-β-actin

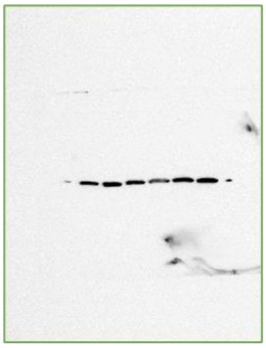

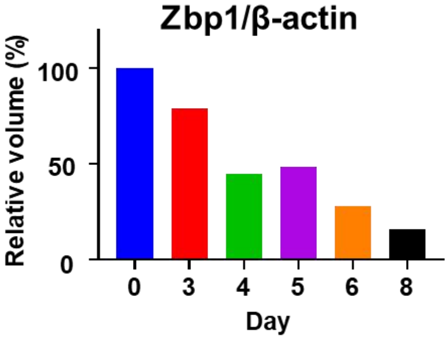

anti-Zbp1

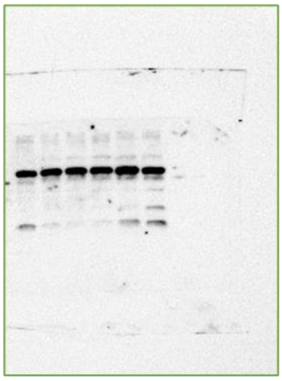

anti-β-actin

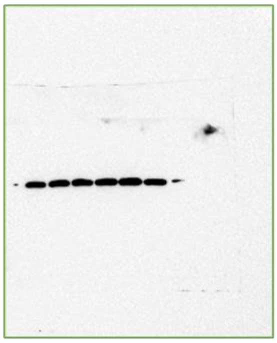

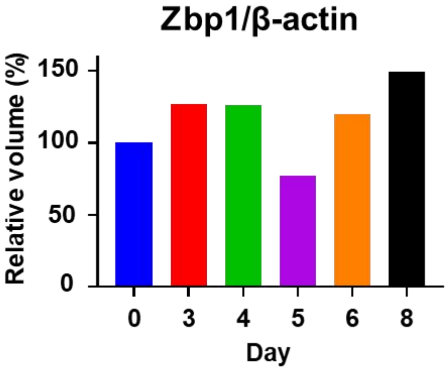

Fig. 4B

anti-Zbp1

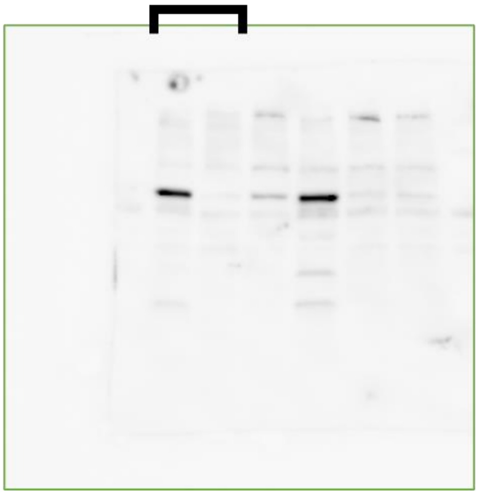

anti-β-actin

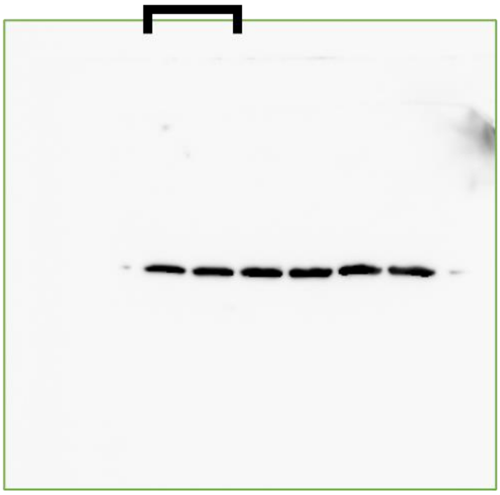

Supplementary Fig S6 cont'd

Fig. 5B

anti-Zbp1

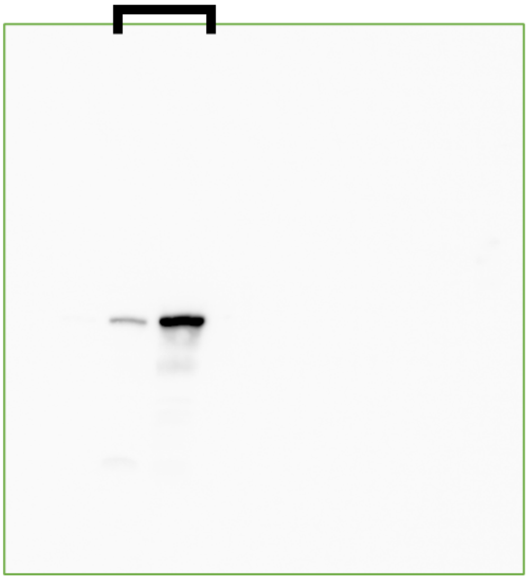

anti-Hprt

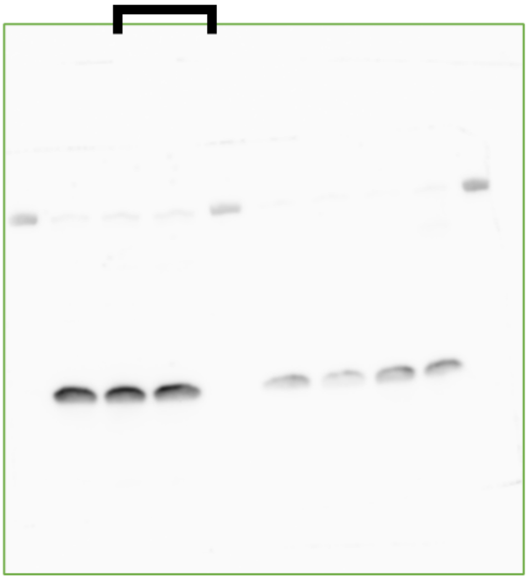

Fig. S2C

anti-Runx2

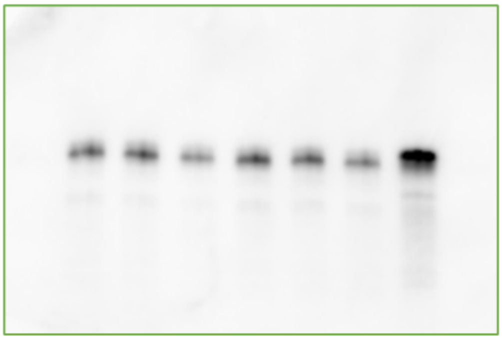

anti-Sp7, Hprt

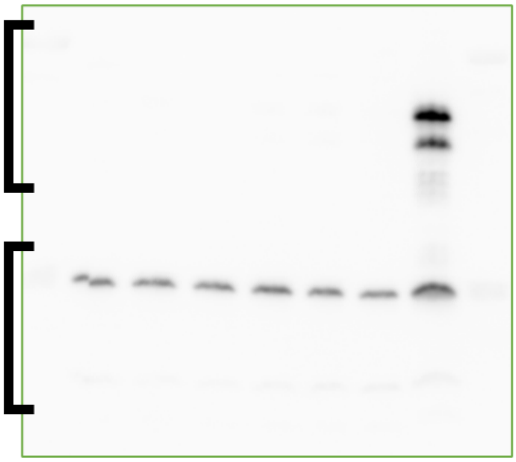

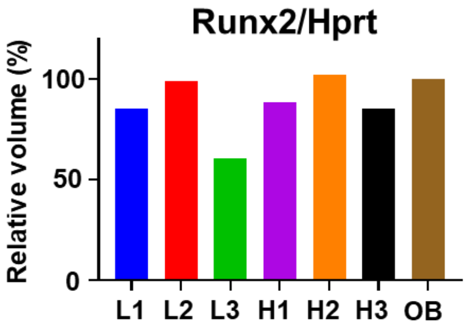

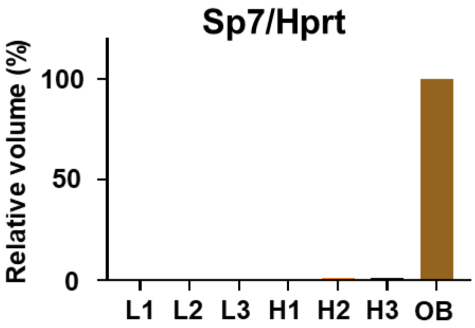

Fig. S5E

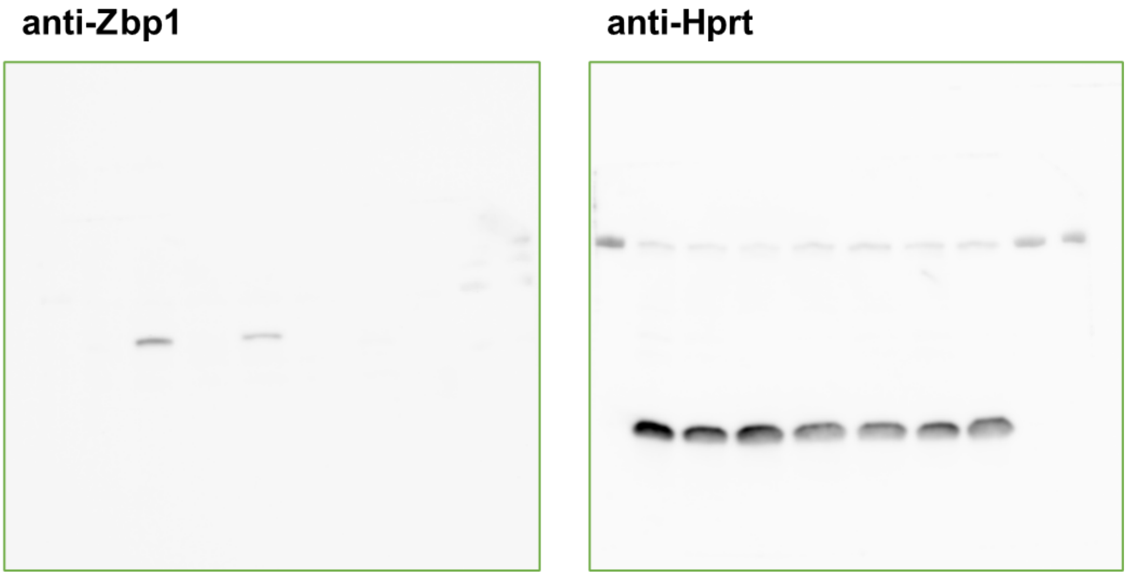
anti-Zbp1
anti-Hprt

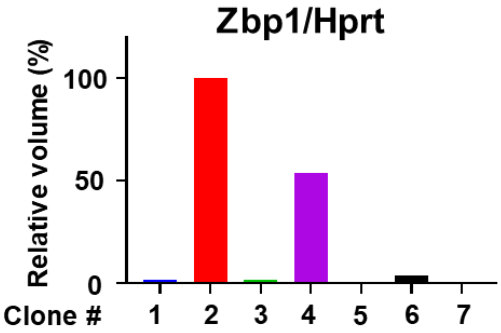
Zbp1/Hprt
Relative volume (%)
100
50
0
Clone #
1
2
3
4
5
6
7

Supplementary Movie S1

Isolation of the murine PDL tissue from the mesial root of the maxillary first molar using a 0.5mm micro-curette. A snapshot of the movie is shown below.

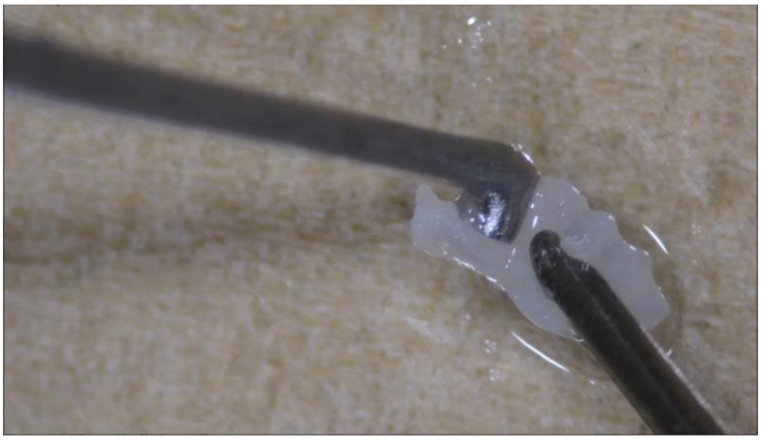

qRT-PCR Primer pairs

Gene name	Accession#	Forward	Reverse
Plap1/Aspn	NM_025711	CCTCTTGAGAACAACGGGATAG	TCCAGCAAAGTTGGTGGTAG
Postn	NM_015784	TGTGTATCGGACGGCTATCT	CTCTGCTGGTTGGATGATTTCT
Scx	NM_198885	AGGGCCTATGAACAGAGAGAT	GTAGAGAGCCAGCATGGAAAG
Runx2	NM_009820	TGGCTTGGGTTTCAGGTTAG	GGTTTCTTAGGGTCTTGGAGTG
Sp7	NM_130458	GGAGACCTTGCTCGTAGATTTC	CAGAGAGACACCCACAGAAAC
Dlx5	NM_010056	GCAGCCAGCTCAATCAATTC	GCAAGAGAAAGTAGCCCATCTA
Zfp521	NM_145492	ACCATCAGCTGTGAGTGTATTT	CAAGGGTGCAGGCATTAGA
Pparg	NM_011146	CTGGCCTCCCTGATGAATAAAG	AGGCTCCATAAAGTCACCAAAG
Zfp423	NM_033327	CCATGAGGCGCTGCTATTAT	CTAGCTGGAGCAGGACAATAAA
Hprt1	NM_013556	GGCCAGACTTTGTTGGATTTG	CGCTCATCTTAGGCTTTGTATTTG
Zbp1	NM_021394	GCCCAGCCTAGCCTTGATGA	GGTGCTTATTTCTCATGGAATACAGG
B2m	NM_009735	GGCCTGTATGCTATCCAGAAAAC	CGTAGCAGTTCAGTATGTTCGG

PCR primer pairs to amplify target region of CRISPR/Cas9 for cloning

Insert (Zbp1 genome)	Forward	ACTCACTATAGGGCGAATTGGTTGGCTCTCCCTCCTTAATCCT
	Reverse	ACTAAAGGGAACAAAAGCTGAAGTGAACCCACTCCTGTATTCCA
vector (pBluescript)	Forward	ATACAGGAGTGGGTTCACTTCAGCTTTTGTTCCCTTTAGTGAGGG
	Reverse	ATTAAGGAGGGAGAGCCAACCAATTCGCCCTATAGTGAGTCG

Seqencing primers

T3	GCAATTAACCCTCACTAAAGG
T7	TAATACGACTCACTATAGGG

Genotyping PCR primers

Zbp1-F	AAGCAGCCAGGTTTCTGGATT
Zbp1-R_KO	TAAAGCGCATGCTCCAGACT
Zbp1-R_WT	GGATTAGATGGTGTAGGGCCAG

Antibodies used in flow cytometry experiments

CD73	BioLegend	127205	PE-conjugated Rat IgG1, κ	1:200
CD105	BioLegend	120407	PE-conjugated Rat IgG2a, κ	1:200
CD140a	BioLegend	135905	PE-conjugated Rat IgG2a, κ	1:200
CD140b	BioLegend	136005	PE-conjugated Rat IgG2a, κ	1:200
Isotype control	BioLegend	400407	PE-conjugated Rat IgG1, κ	1:200
Isotype control	BioLegend	400507	PE-conjugated Rat IgG2a, κ	1:200

Antibodies used in Western blot experiments

Zbp1	AdipoGen	AG-20B-0010-C100	Mouse mAb	1:1,000
Runx2	CST	8486S	Rabbit mAb	1:1,000
Sp7	Abcam	ab22552	Rabbit pAb	1:1,000
Hprt	Abcam	ab10479	Rabbit pAb	1:1,000
β-actin	Sigma	A1978	Mouse mAb	1:10,000
anti-mouse IgG	GE Healthcare	NA931VS	HRP conjugated	1:10,000
anti-rabbit IgG	GE Healthcare	NA934VS	HRP conjugated	1:10,000

Data	GraphPad statistical analysis
Fig. 1B	Unpaired t test
Fig. 2A	Unpaired t test
Fig. 2B	Unpaired t test
Fig. 2C	Unpaired t test
Fig. 2D	Unpaired t test
Fig. 4A	Unpaired t test
Fig. 4C	Two way ANOVA, followed by Tukey's multiple comparisons test*
Fig. 5A	Unpaired t test
Fig. 5C	Two way ANOVA, followed by Sidak's multiple comparisons test*
Fig. S1D	Unpaired t test
Fig. S3B	One way ANOVA, followed by Tukey's multiple comparisons test*

*According to the GraphPad Prism software recommendation

Table Analyzed	**Aspn**
Column B	PDL
vs.	vs.
Column A	gingiva

Unpaired t test

P value	**0.0249**
P value summary	*
Significantly different (P < 0.05)?	Yes
One- or two-tailed P value?	Two-tailed
t, df	t=2.971 df=6

How big is the difference?

Mean ± SEM of column A	1 ± 0.3123, n=4
Mean ± SEM of column B	59.37 ± 19.64, n=4
95% confidence interval	10.3 to 106.4
R squared (eta squared)	0.5954

F test to compare variances

F, DFn, Dfd	3956, 3, 3
P value	<0.0001
P value summary	****
Significantly different (P < 0.05)?	Yes

Table Analyzed	**Postn**
Column B	PDL
vs.	vs.
Column A	gingiva

Unpaired t test

P value	**0.0163**
P value summary	*
Significantly different (P < 0.05)?	Yes
One- or two-tailed P value?	Two-tailed
t, df	t=3.306 df=6

How big is the difference?

Mean ± SEM of column A	1 ± 0.2945, n=4
Mean ± SEM of column B	44.31 ± 13.1, n=4
95% confidence interval	11.25 to 75.37
R squared (eta squared)	0.6456

F test to compare variances

F, DFn, Dfd	1978, 3, 3
P value	<0.0001
P value summary	****
Significantly different (P < 0.05)?	Yes

Table Analyzed	**Scx**
Column B	PDL
vs.	vs.
Column A	gingiva

Unpaired t test

P value	**0.1671**
P value summary	ns
Significantly different (P < 0.05)?	No
One- or two-tailed P value?	Two-tailed
t, df	t=1.572 df=6

How big is the difference?

Mean ± SEM of column A	1 ± 0.5473, n=4
Mean ± SEM of column B	25.01 ± 15.27, n=4
95% confidence interval	-13.37 to 61.4
R squared (eta squared)	0.2917

F test to compare variances

F, DFn, Dfd	778.3, 3, 3
P value	0.0002
P value summary	***
Significantly different (P < 0.05)?	Yes

Table Analyzed	**CD73**
Column B	Data Set-B
vs.	vs.
Column A	Data Set-A

Unpaired t test

P value	**>0.9999**
P value summary	ns
Significantly different (P < 0.05)?	No
One- or two-tailed P value?	Two-tailed
t, df	t=0 df=4

How big is the difference?

Mean ± SEM of column A	22.81 ± 4.77, n=3
Mean ± SEM of column B	22.81 ± 5.084, n=3
95% confidence interval	-19.36 to 19.36
R squared (eta squared)	0

F test to compare variances

F, DFn, Dfd	1.136, 2, 2
P value	0.9361
P value summary	ns
Significantly different (P < 0.05)?	No

Table Analyzed	**CD105**
Column B	Data Set-B
vs.	vs.
Column A	Data Set-A

Unpaired t test

P value	**0.4077**
P value summary	ns
Significantly different (P < 0.05)?	No
One- or two-tailed P value?	Two-tailed
t, df	t=0.9242 df=4

How big is the difference?

Mean ± SEM of column A	18.68 ± 1.593, n=3
Mean ± SEM of column B	17.07 ± 0.7228, n=3
95% confidence interval	-6.473 to 3.24
R squared (eta squared)	0.176

F test to compare variances

F, DFn, Dfd	4.857, 2, 2
P value	0.3415
P value summary	ns
Significantly different (P < 0.05)?	No

Table Analyzed	**CD140a**
Column B	Data Set-B
vs.	vs.
Column A	Data Set-A

Unpaired t test

P value	**0.3193**
P value summary	ns
Significantly different (P < 0.05)?	No
One- or two-tailed P value?	Two-tailed
t, df	t=1.136 df=4

How big is the difference?

Mean ± SEM of column A	58.99 ± 11.97, n=3
Mean ± SEM of column B	39.57 ± 12.2, n=3
Difference between means	-19.42 ± 17.09
95% confidence interval	-66.86 to 28.03
R squared (eta squared)	0.244

F test to compare variances

F, DFn, Dfd	1.04, 2, 2
P value	0.9806
P value summary	ns
Significantly different (P < 0.05)?	No

Table Analyzed	**CD140b**
Column B	Data Set-B
vs.	vs.
Column A	Data Set-A

Unpaired t test

P value	**0.935**
P value summary	ns
Significantly different (P < 0.05)?	No
One- or two-tailed P value?	Two-tailed
t, df	t=0.08675 df=4

How big is the difference?

Mean ± SEM of column A	3.743 ± 0.1584, n=3
Mean ± SEM of column B	3.683 ± 0.6733, n=3
Difference between means	-0.06 ± 0.6917
95% confidence interval	-1.98 to 1.86
R squared (eta squared)	0.001878

F test to compare variances

F, DFn, Dfd	18.08, 2, 2
P value	0.1048
P value summary	ns
Significantly different (P < 0.05)?	No

Table S3 (Fig. 2B)

Table Analyzed	**Supernatant**
Column B	Data Set-B
vs.	vs.
Column A	Data Set-A

Unpaired t test

P value	**0.5349**
P value summary	ns
Significantly different (P < 0.05)?	No
One- or two-tailed P value?	Two-tailed
t, df	t=0.6781 df=4

How big is the difference?

Mean ± SEM of column A	6.532 ± 0.0595, n=3
Mean ± SEM of column B	6.892 ± 0.5277, n=3
95% confidence interval	-1.114 to 1.834
R squared (eta squared)	0.1031

F test to compare variances

F, DFn, Dfd	78.63, 2, 2
P value	0.0251
P value summary	*
Significantly different (P < 0.05)?	Yes

Table Analyzed	**Lysate**
Column B	High
vs.	vs.
Column A	Low

Unpaired t test

P value	**0.2948**
P value summary	ns
Significantly different (P < 0.05)?	No
One- or two-tailed P value?	Two-tailed
t, df	t=1.204 df=4

How big is the difference?

Mean ± SEM of column A	15.22 ± 2.133, n=3
Mean ± SEM of column B	12.47 ± 0.7952, n=3
95% confidence interval	-9.064 to 3.579
R squared (eta squared)	0.2661

F test to compare variances

F, DFn, Dfd	7.198, 2, 2
P value	0.244
P value summary	ns
Significantly different (P < 0.05)?	No

Table Analyzed	Data 1
Column B	Low
vs.	vs.
Column A	High

Unpaired t test

P value	**0.0658**
P value summary	ns
Significantly different (P < 0.05)?	No
One- or two-tailed P value?	Two-tailed
t, df	t=2.513 df=4

How big is the difference?

Mean ± SEM of column A	11.76 ± 1.152, n=3
Mean ± SEM of column B	23.05 ± 4.342, n=3
95% confidence interval	-1.181 to 23.76
R squared (eta squared)	0.6123

F test to compare variances

F, DFn, Dfd	14.2, 2, 2
P value	0.1316
P value summary	ns
Significantly different (P < 0.05)?	No

Table S3 (Fig. 2D)

Table Analyzed	**Runx2/Hprt1**
Column B	low
vs.	vs.
Column A	high

Unpaired t test

P value	**0.2834**
P value summary	ns
Significantly different (P < 0.05)?	No
One- or two-tailed P value?	Two-tailed
t, df	t=1.238 df=4

How big is the difference?

Mean ± SEM of column A	0.7967 ± 0.1013, n=3
Mean ± SEM of column B	1 ± 0.1293, n=3
95% confidence interval	-0.2526 to 0.6593
R squared (eta squared)	0.2771

F test to compare variances

F, DFn, Dfd	1.629, 2, 2
P value	0.7606
P value summary	ns
Significantly different (P < 0.05)?	No

Table Analyzed	**Sp7/Hprt1**
Column B	low
vs.	vs.
Column A	high

Unpaired t test

P value	**0.7225**
P value summary	ns
Significantly different (P < 0.05)?	No
One- or two-tailed P value?	Two-tailed
t, df	t=0.3811 df=4

How big is the difference?

Mean ± SEM of column A	1.129 ± 0.1964, n=3
Mean ± SEM of column B	1 ± 0.2766, n=3
95% confidence interval	-1.071 to 0.8126
R squared (eta squared)	0.03504

F test to compare variances

F, DFn, Dfd	1.984, 2, 2
P value	0.6703
P value summary	ns
Significantly different (P < 0.05)?	No

Table Analyzed	**Dlx5/Hprt1**
Column B	low
vs.	vs.
Column A	high

Unpaired t test

P value	**0.1476**
P value summary	ns
Significantly different (P < 0.05)?	No
One- or two-tailed P value?	Two-tailed
t, df	t=1.792 df=4

How big is the difference?

Mean ± SEM of column A	0.7324 ± 0.1228, n=3
Mean ± SEM of column B	1 ± 0.08489, n=3
95% confidence interval	-0.1469 to 0.6822
R squared (eta squared)	0.4454

F test to compare variances

F, DFn, Dfd	2.094, 2, 2
P value	0.6464
P value summary	ns
Significantly different (P < 0.05)?	No

Table Analyzed	**Pparg/Hprt1**
Column B	low
vs.	vs.
Column A	high

Unpaired t test

P value	**0.6302**
P value summary	ns
Significantly different (P < 0.05)?	No
One- or two-tailed P value?	Two-tailed
t, df	t=0.5204 df=4

How big is the difference?

Mean ± SEM of column A	0.8644 ± 0.1105, n=3
Mean ± SEM of column B	1 ± 0.236, n=3
Difference between means	0.1356 ± 0.2606
95% confidence interval	-0.588 to 0.8592
R squared (eta squared)	0.06342

F test to compare variances

F, DFn, Dfd	4.56, 2, 2
P value	0.3597
P value summary	ns
Significantly different (P < 0.05)?	No

Table Analyzed	**Zfp423/Hprt1**
Column B	low
vs.	vs.
Column A	high

Unpaired t test

P value	**0.6986**
P value summary	ns
Significantly different (P < 0.05)?	No
One- or two-tailed P value?	Two-tailed
t, df	t=0.4163 df=4

How big is the difference?

Mean ± SEM of column A	0.8564 ± 0.1148, n=3
Mean ± SEM of column B	1 ± 0.3253, n=3
Difference between means	0.1436 ± 0.345
95% confidence interval	-0.8143 to 1.102
R squared (eta squared)	0.04152

F test to compare variances

F, DFn, Dfd	8.024, 2, 2
P value	0.2216
P value summary	ns
Significantly different (P < 0.05)?	No

Table Analyzed	**Zfp521/Hprt1**
Column B	low
vs.	vs.
Column A	high

Unpaired t test

P value	**0.8071**
P value summary	ns
Significantly different (P < 0.05)?	No
One- or two-tailed P value?	Two-tailed
t, df	t=0.2608 df=4

How big is the difference?

Mean ± SEM of column A	0.8861 ± 0.1372, n=3
Mean ± SEM of column B	1 ± 0.4146, n=3
Difference between means	0.1139 ± 0.4367
95% confidence interval	-1.099 to 1.326
R squared (eta squared)	0.01673

F test to compare variances

F, DFn, Dfd	9.13, 2, 2
P value	0.1974
P value summary	ns
Significantly different (P < 0.05)?	No

Table Analyzed	Column: Entering averaged data
Column B	Data Set-B
vs.	vs.
Column A	Data Set-A

Unpaired t test

P value	**0.0024**
P value summary	**
Significantly different (P < 0.05)?	Yes
One- or two-tailed P value?	Two-tailed
t, df	t=6.795 df=4

How big is the difference?

Mean ± SEM of column A	1 ± 0.1159, n=3
Mean ± SEM of column B	0.1877 ± 0.02935, n=3
95% confidence interval	-1.144 to -0.4804
R squared (eta squared)	0.9203

F test to compare variances

F, DFn, Dfd	15.59, 2, 2
P value	0.1206
P value summary	ns
Significantly different (P < 0.05)?	No

Table S3 (Fig. 4C)

Table Analyzed	Data 4
Two-way ANOVA	Ordinary
Alpha	0.05

Source of Variation	% of total variation	P value	P value summary	Significant?
Interaction	11.39	**<0.0001**	****	Yes
Row Factor	72.76	**<0.0001**	****	Yes
Column Factor	11.64	**<0.0001**	****	Yes

ANOVA table	SS	DF	MS	F (DFn, DFd)	P value
Interaction	45039	6	7507	F (6, 24) = 10.8	P<0.0001
Row Factor	287726	3	95909	F (3, 24) = 138	P<0.0001
Column Factor	46021	2	23011	F (2, 24) = 33.12	P<0.0001
Residual	16676	24	694.8		

Within each row, compare columns (simple effects within rows)

Number of families	4
Number of comparisons per family	3
Alpha	0.05

**Tukey's multiple comparisons test**	Mean Diff.	95.00% CI of diff.	Significant?	Summary	Adjusted P Value
Row 1					
High vs. Low	2.567	-51.18 to 56.32	No	ns	**0.9922**
High vs. High/Zbp1-KO	1.482	-52.27 to 55.23	No	ns	**0.9974**
Low vs. High/Zbp1-KO	-1.086	-54.83 to 52.66	No	ns	**0.9986**
Row 2					
High vs. Low	36.58	-17.17 to 90.32	No	ns	**0.226**
High vs. High/Zbp1-KO	38.88	-14.87 to 92.63	No	ns	**0.1889**
Low vs. High/Zbp1-KO	2.301	-51.45 to 56.05	No	ns	**0.9937**
Row 3					
High vs. Low	106.3	52.59 to 160.1	Yes	***	**0.0001**
High vs. High/Zbp1-KO	91.69	37.94 to 145.4	Yes	***	**0.0008**
Low vs. High/Zbp1-KO	-14.64	-68.39 to 39.11	No	ns	**0.7771**
Row 4					
High vs. Low	204.7	151 to 258.5	Yes	****	**<0.0001**
High vs. High/Zbp1-KO	50.45	-3.299 to 104.2	No	ns	**0.0687**
Low vs. High/Zbp1-KO	-154.3	-208 to -100.5	Yes	****	**<0.0001**

Test details	Mean 1	Mean 2	Mean Diff.	SE of diff.	N1	N2	q	DF
Row 1								
High vs. Low	14.54	11.97	2.567	21.52	3	3	0.1687	24
High vs. High/Zbp1-KO	14.54	13.06	1.482	21.52	3	3	0.09735	24
Low vs. High/Zbp1-KO	11.97	13.06	-1.086	21.52	3	3	0.07135	24
Row 2								
High vs. Low	72.46	35.88	36.58	21.52	3	3	2.403	24
High vs. High/Zbp1-KO	72.46	33.58	38.88	21.52	3	3	2.555	24
Low vs. High/Zbp1-KO	35.88	33.58	2.301	21.52	3	3	0.1512	24
Row 3								
High vs. Low	177.3	70.97	106.3	21.52	3	3	6.987	24
High vs. High/Zbp1-KO	177.3	85.62	91.69	21.52	3	3	6.025	24
Low vs. High/Zbp1-KO	70.97	85.62	-14.64	21.52	3	3	0.9621	24
Row 4								
High vs. Low	332.1	127.4	204.7	21.52	3	3	13.45	24
High vs. High/Zbp1-KO	332.1	281.6	50.45	21.52	3	3	3.315	24
Low vs. High/Zbp1-KO	127.4	281.6	-154.3	21.52	3	3	10.14	24

Table Analyzed	Data 3
Column B	Data Set-B
vs.	vs.
Column A	Data Set-A

Unpaired t test

P value	**0.0018**
P value summary	**
Significantly different (P < 0.05)?	Yes
One- or two-tailed P value?	Two-tailed
t, df	t=7.389 df=4

How big is the difference?

Mean ± SEM of column A	1 ± 0.1087, n=3
Mean ± SEM of column B	1.892 ± 0.05249, n=3
95% confidence interval	0.5566 to 1.227
R squared (eta squared)	0.9317

F test to compare variances

F, DFn, Dfd	4.284, 2, 2
P value	0.3785
P value summary	ns
Significantly different (P < 0.05)?	No

Table Analyzed	Data 4
**Two-way ANOVA**	Ordinary
Alpha	0.05

Source of Variation	% of total variation	P value	P value summary	Significant?
Interaction	6.664	<0.0001	****	Yes
Row Factor	86.34	<0.0001	****	Yes
Column Factor	4.61	<0.0001	****	Yes

ANOVA table	SS	DF	MS	F (DFn, DFd)	P value
Interaction	4620	4	1155	F (4, 20) = 13.98	P<0.0001
Row Factor	59868	4	14967	F (4, 20) = 181.1	P<0.0001
Column Factor	3196	1	3196	F (1, 20) = 38.68	P<0.0001
Residual	1653	20	82.64		

Mean of lenti-Zbp1	86.67
Mean of lenti-GFP	66.03
Difference between means	20.64
SE of difference	3.319
95% CI of difference	13.72 to 27.57

Data summary	
Number of columns (Column Factor)	2
Number of rows (Row Factor)	5
Number of values	30

Compare each cell mean with the other cell mean in that row

Number of families	1
Number of comparisons per family	5
Alpha	0.05

**Šídák's multiple comparisons test**	Mean Diff.	95.00% CI of diff.	Below threshold?	Summary	Adjusted P Value
lenti-Zbp1 - lenti-GFP					
Row 1	-1.085	-22.14 to 19.97	No	ns	>0.9999
Row 2	0.6289	-20.42 to 21.68	No	ns	>0.9999
Row 3	35.14	14.09 to 56.19	Yes	***	0.0006
Row 4	5.755	-15.30 to 26.81	No	ns	0.9484
Row 5	62.78	41.73 to 83.83	Yes	****	<0.0001

Test details	Mean 1	Mean 2	Mean Diff.	SE of diff.	N1	N2	t	DF
lenti-Zbp1 - lenti-GFP								
Row 1	7.293	8.378	-1.085	7.423	3	3	0.1461	20
Row 2	45.22	44.59	0.6289	7.423	3	3	0.08473	20
Row 3	121.4	86.26	35.14	7.423	3	3	4.734	20
Row 4	94.1	88.35	5.755	7.423	3	3	0.7753	20
Row 5	165.3	102.6	62.78	7.423	3	3	8.458	20

Table Analyzed	Aspn
Column B	MGF
vs.	vs.
Column A	MPDL

Unpaired t test

P value	<0.0001
P value summary	****
Significantly different (P < 0.05)?	Yes
One- or two-tailed P value?	Two-tailed
t, df	t=31.56, df=4

How big is the difference?

Mean of column A	93.87
Mean of column B	1
95% confidence interval	-101.0 to -84.70
R squared (eta squared)	0.996

F test to compare variances

F, DFn, Dfd	853.0, 2, 2
P value	0.0023
P value summary	**
Significantly different (P < 0.05)?	Yes

Data analyzed

Sample size, column A	3
Sample size, column B	3

Table Analyzed	Postn
Column B	MGF
vs.	vs.
Column A	MPDL

Unpaired t test

P value	0.0052
P value summary	**
Significantly different (P < 0.05)?	Yes
One- or two-tailed P value?	Two-tailed
t, df	t=5.527, df=4

How big is the difference?

Mean of column A	4.01
Mean of column B	1
95% confidence interval	-4.523 to -1.498
R squared (eta squared)	0.8842

F test to compare variances

F, DFn, Dfd	299.7, 2, 2
P value	0.0067
P value summary	**
Significantly different (P < 0.05)?	Yes

Data analyzed

Sample size, column A	3
Sample size, column B	3

Table Analyzed	Scx
Column B	MGF
vs.	vs.
Column A	MPDL

Unpaired t test

P value	0.0992
P value summary	ns
Significantly different (P < 0.05)?	No
One- or two-tailed P value?	Two-tailed
t, df	t=2.139, df=4

How big is the difference?

Mean of column A	1.373
Mean of column B	1
95% confidence interval	-0.8573 to 0.1111
R squared (eta squared)	0.5336

F test to compare variances

F, DFn, Dfd	1.101, 2, 2
P value	0.9517
P value summary	ns
Significantly different (P < 0.05)?	No

Data analyzed

Sample size, column A	3
Sample size, column B	3

Table S3 (Fig. S3B)

Table Analyzed	**Zbp1**
Data sets analyzed	A-E

ANOVA summary

F	24.31
P value	<0.0001
P value summary	****
Significant diff. among means (P < 0.05)?	Yes
R squared	0.9153

Brown-Forsythe test

F (DFn, DFd)	1.407 (4, 9)
P value	0.3069
P value summary	ns
Are SDs significantly different (P < 0.05)?	No

Bartlett's test

Bartlett's statistic (corrected)	
P value	
P value summary	
Are SDs significantly different (P < 0.05)?	

ANOVA table	SS	DF	MS	F (DFn, DFd)	P value
Treatment (between columns)	12.73	4	3.182	F (4, 9) = 24.31	P<0.0001
Residual (within columns)	1.178	9	0.1309		
Total	13.91	13			

Data summary

Number of treatments (columns)	5
Number of values (total)	14

Number of families	1
Number of comparisons per family	10
Alpha	0.05

**Tukey's multiple comparisons test**	Mean Diff.	95.00% CI of diff.	Below thres	Summary	Adjusted P	Value
High vs. Low	1.739	0.7461 to 2.733	Yes	**	0.0016	A-B
High vs. mBMSC	-0.2272	-1.221 to 0.7661	No	ns	0.9334	A-C
High vs. mADSC	1.845	0.8521 to 2.839	Yes	**	0.0011	A-D
High vs. mDPSC	1.845	0.7346 to 2.956	Yes	**	0.0024	A-E
Low vs. mBMSC	-1.967	-2.960 to -0.9733	Yes	***	0.0007	B-C
Low vs. mADSC	0.1061	-0.8873 to 1.099	No	ns	0.9958	B-D
Low vs. mDPSC	0.1057	-1.005 to 1.216	No	ns	0.9973	B-E
mBMSC vs. mADSC	2.073	1.079 to 3.066	Yes	***	0.0004	C-D
mBMSC vs. mDPSC	2.072	0.9618 to 3.183	Yes	**	0.001	C-E
mADSC vs. mDPSC	-0.000312	-1.111 to 1.110	No	ns	>0.9999	D-E

Test details	Mean 1	Mean 2	Mean Diff.	SE of diff.	n1	n2	q	DF
High vs. Low	1.87	0.1303	1.739	0.2954	3	3	8.327	9
High vs. mBMSC	1.87	2.097	-0.2272	0.2954	3	3	1.088	9
High vs. mADSC	1.87	0.02424	1.845	0.2954	3	3	8.835	9
High vs. mDPSC	1.87	0.02455	1.845	0.3303	3	2	7.901	9
Low vs. mBMSC	0.1303	2.097	-1.967	0.2954	3	3	9.415	9
Low vs. mADSC	0.1303	0.02424	0.1061	0.2954	3	3	0.5077	9
Low vs. mDPSC	0.1303	0.02455	0.1057	0.3303	3	2	0.4528	9
mBMSC vs. mADSC	2.097	0.02424	2.073	0.2954	3	3	9.923	9
mBMSC vs. mDPSC	2.097	0.02455	2.072	0.3303	3	2	8.874	9
mADSC vs. mDPSC	0.02424	0.02455	-0.000312	0.3303	3	2	0.001336	9

Fig. 3D anti-β-actin (-)

Lane	Band No.	Adj. Volume (Int)
1	1	2333643
2	1	3175203
3	1	2454748
4	1	1894918
5	1	3762816
6	1	5761511

Fig. 3D anti-β-actin (+)

Lane	Band No.	Adj. Volume (Int)
1	1	11285502
2	1	11714627
3	1	12319903
4	1	16196210
5	1	18908160
6	1	10853852

Fig. 3D anti-Zbp1 (-)

Lane	Band No.	Adj. Volume (Int)
1	1	4733716
2	1	5095272
3	1	2220348
4	1	1869712
5	1	2138448
6	1	1846156

Zbp1/β-actin	Normalized to day0
2.02846622	**100%**
1.60470748	**79%**
0.90451158	**45%**
0.98669811	**49%**
0.56831054	**28%**
0.32042914	**16%**

Fig. 3D anti-Zbp1 (+)

Lane	Band No.	Adj. Volume (Int)
1	1	6921936
2	1	9141387
3	1	9549680
4	1	7681694
5	1	13895373
6	1	9938040

Zbp1/β-actin	Normalized to day0
0.61334764	**100%**
0.78033957	**127%**
0.77514247	**126%**
0.4742896	**77%**
0.73488764	**120%**
0.91562332	**149%**

Fig. 4B anti-Zbp1

Lane	Band No.	Adj. Volume (Int)
1	1	26132590
2	1	703065

Fig. 4B anti-β-actin

Lane	Band No.	Adj. Volume (Int)
1	1	38132738
2	1	43977538

Zbp1/β-ac	Normalized to control
0.685306	**100%**
0.015987	**2%**

Fig. 5B anti-Zbp1

Lane	Band No.	Adj. Volume (Int)
1	1	8056496
2	1	63863240

Fig. 5B anti-Hprt

Lane	Band No.	Adj. Volume (Int)
1	1	37735656
2	1	37340569

Zbp1/β-ac	Normalized to control
0.213498	**100%**
1.710291	**801%**

Fig. S4E anti-Zbp1

Lane	Band No.	Adj. Volume (Int)
1	1	187715
2	1	8565150
3	1	161645
4	1	3688905
5	1	24585
6	1	222750
7	1	10890

Zbp1/Hprt	Normalized to #2
0.003339	**2%**
0.200254	**100%**
0.00317	**2%**
0.107307	**54%**
0.000843	**0%**
0.007081	**4%**
0.000327	**0%**

Fig. S4E anti-Hprt

Lane	Band No.	Adj. Volume (Int)
1	1	56223104
2	1	42771404
3	1	50990848
4	1	34377097
5	1	29171186
6	1	31457684
7	1	33335808

Fig. S2C anti-Sp7

Lane	Band No.	Adj. Volume (Int)
1	1	11658
1	2	11136
2	1	16356
2	2	18096
3	1	24302
3	2	16588
4	1	87000
4	2	23954
5	1	214832
5	2	124120
6	1	241570
6	2	153758
7	1	52267520
7	2	29190784

per lane	Sp7/Hprt	Normalized to control
11397	0.000741	**0%**
17226	0.001212	**0%**
20445	0.00148	**0%**
55477	0.003318	**0%**
169476	0.012855	**1%**
197664	0.016172	**1%**
40729152	1.300324	**100%**

Runx2/Hprt	Normalized to control
1.82633845	**85%**
2.12090842	**99%**
1.30135844	**61%**
1.89892375	**88%**
2.19921506	**102%**
1.82874734	**85%**
2.14791686	**100%**

Fig. S2C anti-Hprt

Lane	Band No.	Adj. Volume (Int)
1	1	15390822
2	1	14211566
3	1	13813454
4	1	16717804
5	1	13184154
6	1	12222514
7	1	31322304

Fig. S2C anti-Runx2

Lane	Band No.	Adj. Volume (Int)
1	1	28108850
2	1	30141430
3	1	17976255
4	1	31745835
5	1	28994790
6	1	22351890
7	1	67277705
